# Development‐based In Vivo Bioreactor Strategy for Challenging Senescent Bone Reconstruction

**DOI:** 10.1002/advs.202522408

**Published:** 2026-03-09

**Authors:** Wenchao Zhang, Kai Dai, Tong Shen, Zehua Gao, Fengying Yan, Yuke Feng, Xuanlin Wang, Shaozhen Zhang, Jing Wang, Changsheng Liu

**Affiliations:** ^1^ State Key Laboratory of Bioreactor Engineering East China University of Science and Technology Shanghai China; ^2^ Engineering Research Center for Biomedical Materials of the Ministry of Education East China University of Science and Technology Shanghai China; ^3^ Frontiers Science Center for Materiobiology and Dynamic Chemistry East China University of Science and Technology Shanghai China; ^4^ Key Laboratory for Ultrafine Materials of Ministry of Education East China University of Science and Technology Shanghai China

**Keywords:** in vivo bioreactor, developmental engineering, segmental bone defect, aging

## Abstract

Critical segmental bone defects in elderly patients pose a formidable clinical challenge due to limited autograft availability, senescent bone dysfunction, and compromised healing from fibrous tissue invasion. Here, we present a development‐based in vivo bioreactor strategy wherein BMP‐2‐loaded biomaterials trigger the body's intrinsic developmental programs, using the organism as a bioreactor to engineer bone. Distinct from classical developmental engineering, this in vivo bioreactor‐derived bone (vBR‐Bone) recapitulates native osseous architecture, including vasculature, cortical bone, trabeculae, and bone marrow niche. In aged murine models, the vBR‐Bone exhibits a rejuvenated restoration of bone bioactivity lost in aging, with reduced senescence, elevated remodeling, and improved stem cell functionality. Capitalizing on its restored remodeling capacity of high bone turnover, the vBR‐Bone fragments enclosed in an asymmetric biomimetic periosteum achieved 6‐week repair of critical‐sized 1/3 femoral shaft segmental defects. Through a “compartmentalized” approach that partitions the defect into manageable fragments, vBR‐Bone progressively remodeled and integrated into functional trabecular bone, ultimately restoring bone mineral density, volume, and microstructure in defects of aged mice. The biomimetic periosteum inhibits fibrous invasion while permitting vascular ingrowth, thereby creating a space for regeneration. Mechanistically, the multifactors within vBR‐Bone reconstitute a bone‐remodeling microenvironment, wherein matrix‐released TGF‐β1 activates the PI3K/AKT/mTOR signaling axis via TRAF6‐dependent ubiquitylation to promote robust osteogenesis. This strategy overcomes autograft shortage and senescence‐associated dysfunction, offering a clinically translatable solution for critical age‐related segmental bone defects.

## Graphical abstract

We present a development‐based in vivo bioreactor strategy to generate rejuvenated bone grafts (vBR‐Bone) within aged hosts. By enclosing vBR‐Bone fragments within an asymmetric biomimetic periosteum, segmental femoral defects in aged mice were successfully repaired within 6 weeks. Mechanistically, the multifactors of vBR‐Bone reconstitute a remodeling microenvironment, wherein matrix‐released TGF‐β1 activates the PI3K/AKT/mTOR signaling axis via TRAF6‐dependent ubiquitylation. This mechanism promotes robust osteogenesis and integration, thereby overcoming senescence‐associated healing compromises.

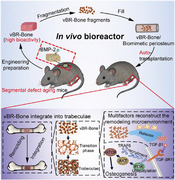

## 1 Introduction

Critical segmental bone defects resulting from high‐energy trauma, surgical debridement, or tumor resection remain a persistent global orthopedic challenge, particularly in older adults, where aging‐associated osteoporosis and fragility fractures exacerbate defect incidence [1, 2, 3, 4]. These defects exceed intrinsic regenerative capacity, especially in the elderly, as organismal senescence impairs tissue function and diminishes cellular support for repair [5, 6, 7, 8]. Autografts face limited availability and declining quality in aged donors, elevating risks for large‐defect reconstruction [9, 10]. Furthermore, fibrous tissue outcompetes osteogenic cells to occupy defect spaces, inhibiting bone healing and frequently causing non‐union complications [11, 12, 13]. These interlocking hurdles render age‐related critical segmental bone defects profoundly difficult to heal.

Autologous bone transplantation remains the gold standard for critical‐sized defects by leveraging bone's inherent remodeling capacity [14, 15, 16], yet suffers from donor site morbidity, limited availability, and age‐related declines in bioactivity that compromise elderly repair [17, 18, 19]. Alternative techniques like Masquelet and Ilizarov have gained clinical traction but remain flawed: Masquelet still requires autologous bone grafts at Stage II [20, 21], while Ilizarov imposes prolonged treatment, early weight‐bearing restrictions, and high nonunion rates [22, 23, 24]. Similarly, biological barrier membranes—engineered to exclude non‐osteogenic cells and prevent fibrous tissue invasion—suffer from insufficient bone mass for structural reconstruction, as well as inadequate osteoinductivity and vascular permeability, thereby limiting their efficacy in repairing large segmental defects [25, 26, 27]. Thus, existing therapeutic approaches fail to address the challenges of repairing excessively large defect volumes, the scarcity of autologous bone supply, and the compromised bioactivity of senescent autologous bone.

Developmental engineering employs principles of embryonic development and tissue regeneration to direct tissue/organ formation, leveraging cell self‐assembly and gene activation to mimic natural organogenesis [28, 29, 30, 31]. The traditional paradigm emphasizes autonomous cellular behavior to replicate developmental cascades. Current cutting‐edge trends in tissue regeneration focus on culturing organoids for damaged tissue replacement via in vitro developmental simulation [32, 33, 34, 35]. Yet in vitro‐derived organoids cannot fully recapitulate the complexity of in vivo tissues, lacking structural integrity and multicellular interactions despite forming 3D cellular networks [36, 37], and facing hurdles such as vascular insufficiency that triggers central necrosis and compromised maturation [35, 38, 39]. Hence, effective strategies for age‐related critical bone defects are urgently warranted.

We develop an innovative technique: a development‐based in vivo bioreactor strategy, defined as a living system that conceptualizes organisms as self‐regulating bioreactors for in vivo bone organogenesis. Bone Morphogenetic Protein‐2 (BMP‐2) effectively stimulates both osteogenesis and chondrogenesis [40, 41, 42]. Guided by developmental engineering principles, we utilize the body as a bioreactor, deploying BMP‐2‐loaded biomaterials to trigger the body's natural developmental programs, initiating a cascade including mesenchymal cell condensation, endochondral ossification, bone remodeling, and bone marrow formation. We engineer in vivo bioreactor‐derived bone (vBR‐Bone) by controlling the developmental timing. Even in aged mice, vBR‐Bone exhibits a rejuvenated restoration of bone bioactivity lost in aging, characterized by reduced cellular senescence, enhanced remodeling, and superior stem cell functionality. Capitalizing on its remodeling capacity of high bone turnover, the vBR‐Bone graft efficiently repairs a 1/3 femoral shaft segmental defect within 6 weeks via a compartmentalized “remodeling and integration” approach, significantly enhancing treatment efficiency. Mechanistically, vBR‐Bone‐derived multifactors reconstruct the bone remodeling microenvironment, where matrix‐released TGF‐β1 activated the PI3K/AKT/mTOR axis through TRAF6‐dependent ubiquitylation, driving robust osteogenesis. This strategy effectively addresses the triad of challenges posed by: the limited availability of therapeutic autologous bone, its compromised bioactivity in a senescent state, and the reconstruction of weight‐bearing femoral segmental defects.

## 2 Results

### 2.1 In Vivo Bioreactor Emerges from a BMP‐2‐trigger Developmental Cascade

**FIGURE 1:**
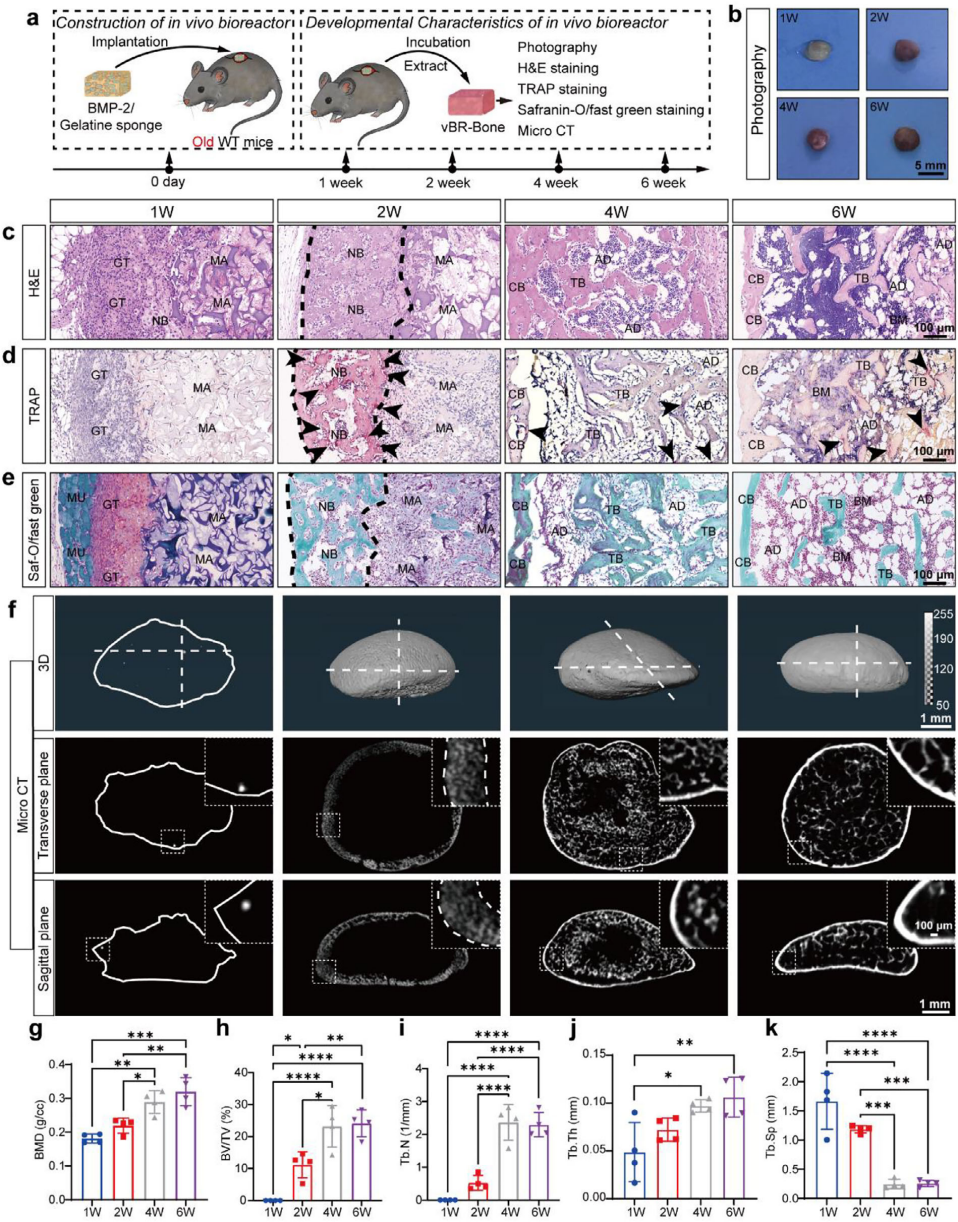
BMP‐2‐loaded biomaterials initiate a bone developmental cascade to construct an in vivo bioreactor. (a) Schematic illustration of the construction process and characterization of the in vivo bioreactor‐derived bone (vBR‐Bone). (b) Photography of vBR‐Bone generated by the in vivo bioreactor. (c–e) Histological staining, including H&E, TRAP, and Saf‐O/fast green staining. By the second week, vBR‐Bone exhibited endochondral ossification (c,e) and active osteoclastic resorption (d). GT: granulation tissue, NB: new bone, MU: muscle, MA: material, CB: cortical bone, Tb: trabecula, AD: adipocytes, BM: bone marrow. Black arrows indicate TRAP‐positive areas. f‐k, Micro‐CT 3D/2D images and quantitative analysis of vBR‐Bone (N = 4). During the 2W–4 W developmental stage, vBR‐Bone showed peak increase in bone mineral density and relative bone volume (g,h), corresponding to a rapid developmental phase. Scale bar: 5 mm (b), 1 mm (f), and 100 µm (c–e, and magnified images of (f). Color bar for 3D reconstruction: 50–255. Color bar for 2D cross‐sections: 35–156. Data are presented as the mean ± SD. ^*^ *p* < 0.05, ^**^ *p* < 0.01, ^***^ *p* < 0.001, ^****^ *p* < 0.0001, ordinary one‐way ANOVA followed by Tukey's multiple comparisons test.

BMP‐2, a key member of the TGF‐β superfamily, drives bone formation through endochondral ossification [40, 41, 43]. BMP‐2‐loaded gelatin sponges were subcutaneously implanted on the dorsum of aged mice to establish the in vivo bioreactor and the developmental characteristics of the bioreactor‐engineered bone were evaluated across sequential time points (Figure 1a,b). By the first week, cellular infiltration into the outer scaffold region was observed, accompanied by the formation of granulation tissue and chondrocyte‐like cells, alongside initial bone deposition. By the second week, cartilage matured into bone tissue followed by active resorption. By the fourth week, new bone had developed into cortical and trabecular structures concurrent with scaffold degradation. By the sixth week, mature bone marrow cavities had formed, populated by hematopoietic cells and adipocytes (Figure 1c–e). Given that the resulting tissue recapitulated the architecture of native femur (Figure S1a–c), including cortical bone, trabeculae, and bone marrow niche, we termed this biomanufactured authentic bone‐like tissue “in vivo bioreactor‐derived bone (vBR‐Bone)”. This progression demonstrated that vBR‐Bone development occurred through sequential phases: fibrous proliferation, endochondral ossification, bone remodeling, and marrow formation. Notably, active bone resorption was observed during endochondral ossification, demonstrating robust osteoclastic activity and indicating that this phase corresponds to the active bone remodeling period.

Micro‐CT reconstruction enabled vBR‐Bone interior visualization through sagittal and transverse planes along the white dotted line in 3D images (Figure 1f). By the second week, new bone formation was detected at the periphery of the vBR‐Bone, while the interior predominantly remained composed of scaffold material. Between weeks 2 and 4, vBR‐Bone underwent rapid development with robust trabecular formation. During this period, the most pronounced increases occurred in bone mineral density (BMD), bone volume/total volume (BV/TV), and trabecular number (Tb.N), while trabecular separation (Tb.Sp) exhibited the sharpest decline (Figure 1g–k). This accelerated progression indicated vBR‐Bone entered a rapid developmental phase by the second week.

Together, we construct an in vivo bioreactor by triggering developmental cascades with BMP‐2‐loaded biomaterials to generate real bone tissue—vBR‐Bone. At the critical two‐week phase, cartilage‐to‐bone transition coincides with resorptive activity, marking a rapid developmental window that enables effective replacement of senescent autologous bone for bone repair.

### 2.2 Engineered vBR‐Bone Exhibits Restoration of Bone Bioactivity Lost during Aging

**FIGURE 2:**
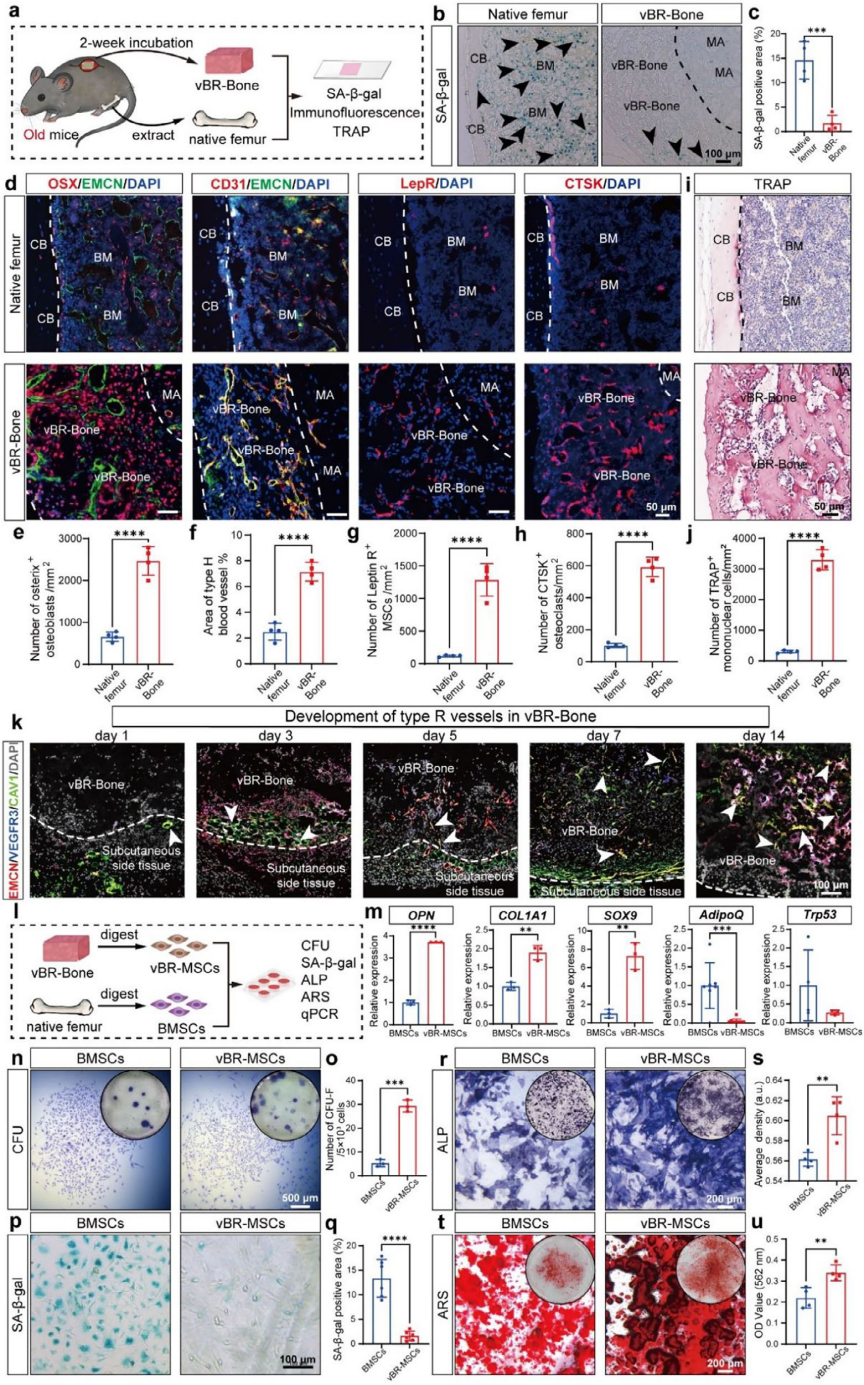
In vivo bioreactor‐derived bone (vBR‐Bone) exhibits restoration of bone bioactivity lost in aging. (a) Schematic illustration of histological evaluation of in vivo bioreactor‐engineered vBR‐Bone. (b,c) SA‐β‐gal staining and quantification. vBR‐Bone at 2 W contained fewer senescent cells compared with autologous femurs. Black arrows: senescent cells. (d–h) Immunofluorescence images and quantification of OSX^+^ osteoprogenitor cells, CD31^hi^EMCN^hi^ type H vessels, LepR^+^ MSCs, and CTSK^+^ osteoclasts in 2 W vBR‐Bone. vBR‐Bone at 2 W showed a higher abundance of OSX^+^ osteoprogenitor cells, CD31^hi^EMCN^hi^ type H vessels, LepR^+^ MSCs, and CTSK^+^ osteoclasts compared with autologous femurs (N = 4). NB: new bone, CB: cortical bone, MA: material, BM: bone marrow. (i,j) TRAP staining and its quantification. vBR‐Bone at 2 W showed a higher abundance of TRAP^+^ mononuclear cells (N = 4). (k) Immunofluorescence images of EMCN^+^CAV1^+^VEGFR3^−^ type R vessels depicting the dynamic developmental process of type R vessels in vBR‐Bone. White arrows: type R vessels. (l) Schematic illustration of functional assessment of in vivo bioreactor‐derived MSCs (vBR‐MSCs). (m) qPCR analysis of vBR‐MSCs (N = 3‐6). (n–u), Evaluation and quantification of CFU, SA‐β‐gal staining, ALP staining, and ARS staining (N = 3‐5). vBR‐MSCs exhibited stronger stemness, lower senescence levels, and enhanced osteogenic differentiation compared with BMSCs. Scale bar: 500 µm (n), 200 µm (r,t), 100 µm (b,k,p), 50 µm (d,i). Data are presented as the mean ± SD. ^**^ *p* < 0.01, ^***^ *p* < 0.001, ^****^ *p* < 0.0001, unpaired, two‐tailed Student's t test.

To evaluate the therapeutic potential of vBR‐Bone as an alternative to senescent autologous bone, a comparative analysis of their bioactivity profiles was performed. Targeting the rapid growth phase at 2 weeks, we controlled the in vivo bioreactor development time in aged mice to harvest 2‐week vBR‐Bone for tissue characteristic comparison with the autologous femur (Figure 2a). vBR‐Bone displayed a reduced population of senescent cells (Figure 2b,c) alongside an expanded pool of LepR^+^ MSCs (Figure 2d–g), underscoring its rejuvenated cellular microenvironment. Osteoprogenitor cells (OSX^+^) and type H vessels (CD31^high^EMCN^high^), which are markedly reduced in aging organisms [44, 45], were abundantly enriched in vBR‐Bone, indicating elevated osteogenic activity (Figure 2d–f). Distinct from conventional developmental engineering approaches plagued by insufficient vascularization, the abundant type H vessels confirmed successful recapitulation of a functional vascular system in vBR‐Bone. Together, these results demonstrate that vBR‐Bone exhibits higher osteogenic capacity than senescent autologous femur.

Contrary to the traditional view of increased osteoclastic activity in aging, recent studies reported reduced osteoclast numbers and bone turnover in aged mice [46, 47, 48, 49]. vBR‐Bone contained increased TRAP^+^ mononuclear pre‐osteoclasts and CTSK^+^ osteoclasts, reflecting rejuvenated resorptive activity (Figure 2d, h–j). As specialized post‐arteriolar capillaries in cancellous bone, Type R vessels (expressing *EMCN*, *CAV1*, and *C1qtnf9* but lacking *VEGFR3*) critically regulate bone remodeling by spatially coupling bone‐forming/resorbing cells and secreting niche factors, thereby serving as essential components of the bone remodeling microenvironment [50]. Tracking type R vessel dynamics in developing vBR‐Bone revealed EMCN^+^CAV1^+^VEGFR3^−^ vessels: initially localized subcutaneously by day 1, migrating toward vBR‐Bone by days 3–5, and fully infiltrating them by days 7–14 (Figure 2k). This spatiotemporal progression correlated with sustained high remodeling activity. Together, concomitant ingrowth of type R vessels and enrichment of osteoclasts during vBR‐Bone development co‐generate a high‐turnover microenvironment, reversing age‐related resorption decline to achieve youth‐like remodeling.

To assess the functionality of stem cells, we isolated in vivo bioreactor‐derived MSCs (vBR‐MSCs) from 2‐week vBR‐Bone and bone marrow mesenchymal stem cells (BMSCs) from femurs of aged mice (Figure 2l). vBR‐MSCs exhibited preferential expression of osteogenic (*OPN*, *COL1A1*) and chondrogenic genes (*SOX9*) while downregulating adipogenic (*AdipoQ*) and senescence (*Trp53*) markers (Figure 2m). The CFU count was significantly higher in vBR‐MSCs than in BMSCs, indicating stronger in vitro self‐renewal ability (Figure 2n,o, Figure S2a,b). Lower levels of senescence were shown in vBR‐MSCs, and even after passage to the third generation, the number of SA‐β‐gal–positive cells remained lower than that in BMSCs (Figure 2p,q, Figure S2c,d). Specifically, we observed that vBR‐Bone generated in aged mice contained fewer SA‐β‐gal–positive cells than the young native femur at the tissue level (Figure S2e,f). At the cellular level, vBR‐MSCs exhibited a comparable senescence level to BMSCs isolated from young mice (Young‐BMSCs) during in vitro culture (Figure S2g,h). Alkaline phosphatase (ALP) and Alizarin Red S (ARS) staining confirmed superior osteogenic differentiation in vBR‐MSCs (Figure 2r–u). Together, these findings indicate that vBR‐Bone is significantly superior to senescent bone in maintenance of stem cell function, possessing more efficient bone formation potential.

In summary, the engineered vBR‐Bone exhibits rejuvenated restoration of bone bioactivity lost in aging, indicating that even aged organisms can produce highly active, sufficient, youth‐like bone tissue. This restoration includes the enrichment of MSCs, osteoblasts, and osteoclasts; the maintenance of high‐density type H and type R vasculature; and the subsequent augmentation of both osteogenic and osteoclastic activities. Collectively, these effects enable bone turnover levels that surpass the inefficient remodeling state of senescent bone.

### 2.3 Biomimetic Periosteum Enhances Osteogenesis and Restricts Fibroblast Invasion to Facilitate Bone Regeneration

**FIGURE 3:**
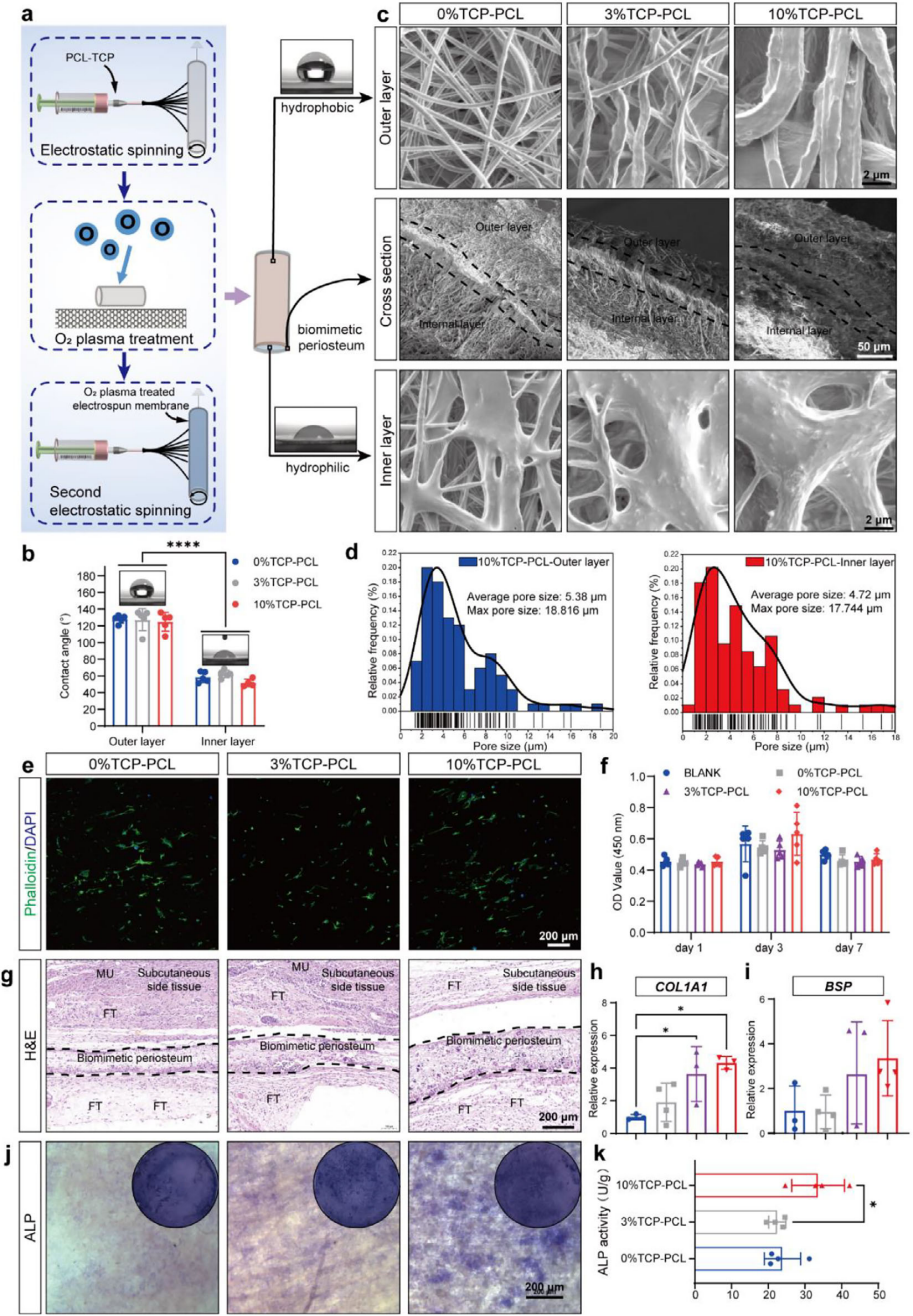
The inner layer of the asymmetric biomimetic periosteum enhances osteogenesis while the outer layer restricts fibroblast infiltration. (a) Fabrication process of the asymmetric bilayer biomimetic periosteum. (b) Contact angle measurements (N = 5). The inner layer of biomimetic periosteum is hydrophilic, and the outer layer is hydrophobic. (c) SEM images of the inner layer, outer layer, and cross‐section of biomimetic periosteum. (d) Frequency distribution of pore sizes in the inner and outer layers. (e) Phalloidin‐FITC fluorescence imaging to evaluate cell adhesion on biomimetic periosteum doped with different TCP concentrations. (f) CCK‐8 assay for cytotoxicity of biomimetic periosteum (N = 5). (g) H&E staining. The subcutaneously implanted biomimetic periosteum exhibited excellent biocompatibility. (h,i) Transcription levels of osteogenic differentiation‐related genes (*COL1A1*, *BSP*) in MSCs cultured on biomimetic periosteum with different TCP concentrations (N = 3–4). (j,l) ALP staining and quantification after osteogenic induction of vBR‐MSCs on biomimetic periosteum with different TCP concentrations (N = 3). Scale bar: 200 µm (e,g,j), 50 µm for cross‐sectional SEM images, and 2 µm for surface SEM images (c). Data are presented as the mean ± SD. ^*^ *p* < 0.05, ^****^ *p* < 0.0001, ordinary one‐way ANOVA (h,i,k) and two‐way ANOVA (f) followed by Tukey's multiple comparisons test.

Critical defects in long bone segments, particularly in elderly individuals, are highly prone to fibrous tissue invasion, which often leads to nonunion (Figure S1d). To block fibrous invasion and encapsulate vBR‐Bone, we fabricated an asymmetric biomimetic periosteum mimicking native periosteum barrier functions. Polycaprolactone (PCL)/tricalcium phosphate (TCP) solutions were electrospun into bilayers: oxygen plasma‐treated inner layer and hydrophobic outer layer (Figure 3a,b). Oxygen plasma treatment converted hydrophobic PCL to hydrophilic [51], enabling fabrication of an asymmetric bilayer biomimetic periosteum with hydrophilicity‐graded layers. The membrane exhibited a dense structure, with the outer layer in filamentous form and the inner layer in a spread configuration. A clear boundary existed at the interface between the inner and outer layers (Figure 3c). Hydrophilic treatment did not significantly alter the distribution or longest diameter of irregular pores in both layers, which remained smaller than fibroblasts (20–40 µm) to delay fibrous invasion (Figure 3d; Figure S3d). As capillary dimensions are approximately 8 µm, the biomimetic periosteum max pore size was 18.28 ± 0.536 um, allowing vascular ingrowth. EDS spectra (Figure S5a–c) showed that the contents of P and Ca elements increase with TCP concentration. These results confirmed the successful fabrication of TCP‐doped biomimetic periosteum.

Contact angles decreased progressively with plasma treatment duration. Given that 50–70° angles favor cell adhesion [52], a 1‐min treatment was chosen (Figure S4a). The contact angle of the untreated outer membrane was obtuse, indicating hydrophobicity, while plasma treatment for 1 min reduced the contact angle to an acute value, confirming hydrophilicity of the inner layer (Figure 3b). Stress‐strain curves revealed two breaking points in 3% and 10% TCP‐PCL biomimetic periosteums, suggesting asymmetrical tensile strength between the inner and outer layers (Figure S4b–d). With increasing TCP concentration, both tensile strength and elongation at break decreased, presumably due to inorganic particle doping.

MSCs were seeded onto the hydrophilic surface of the biomimetic periosteum for culture, with cells adhering to the membrane surface and exhibiting favorable growth morphology (Figure 3e; Figure S4e). CCK‐8 assays demonstrated no cytotoxicity (Figure 3f). Subcutaneous implantation of the biomimetic periosteum on the dorsal side of mice demonstrated favorable in vivo biocompatibility, characterized by the absence of significant inflammatory responses and delayed fibroblast infiltration (Figure 3g). The biomimetic periosteum with a 10% TCP content promoted the expression of osteogenic markers [53], including Collagen I (*COL1A1*) and bone sialoprotein (*BSP*) (Figure 3h,i). The 10% TCP‐PCL biomimetic periosteum exhibited larger ALP staining areas and higher ALP enzyme activity, confirming that 10% TCP‐PCL was more conducive to promoting osteogenesis (Figure 3j,k).

Together, we fabricate an asymmetric biomimetic periosteum via electrospinning with a dense structure capable of blocking fibrous invasion. The biomimetic periosteum features a hydrophilic inner layer that facilitated cell adhesion and osteogenic differentiation, and a hydrophobic outer layer that further served as a barrier to fibrous tissues, thereby providing a regenerative space for vBR‐Bone.

### 2.4 vBR‐Bone Repairs Segmental Defects through a Compartmentalized Remodeling Approach

**FIGURE 4:**
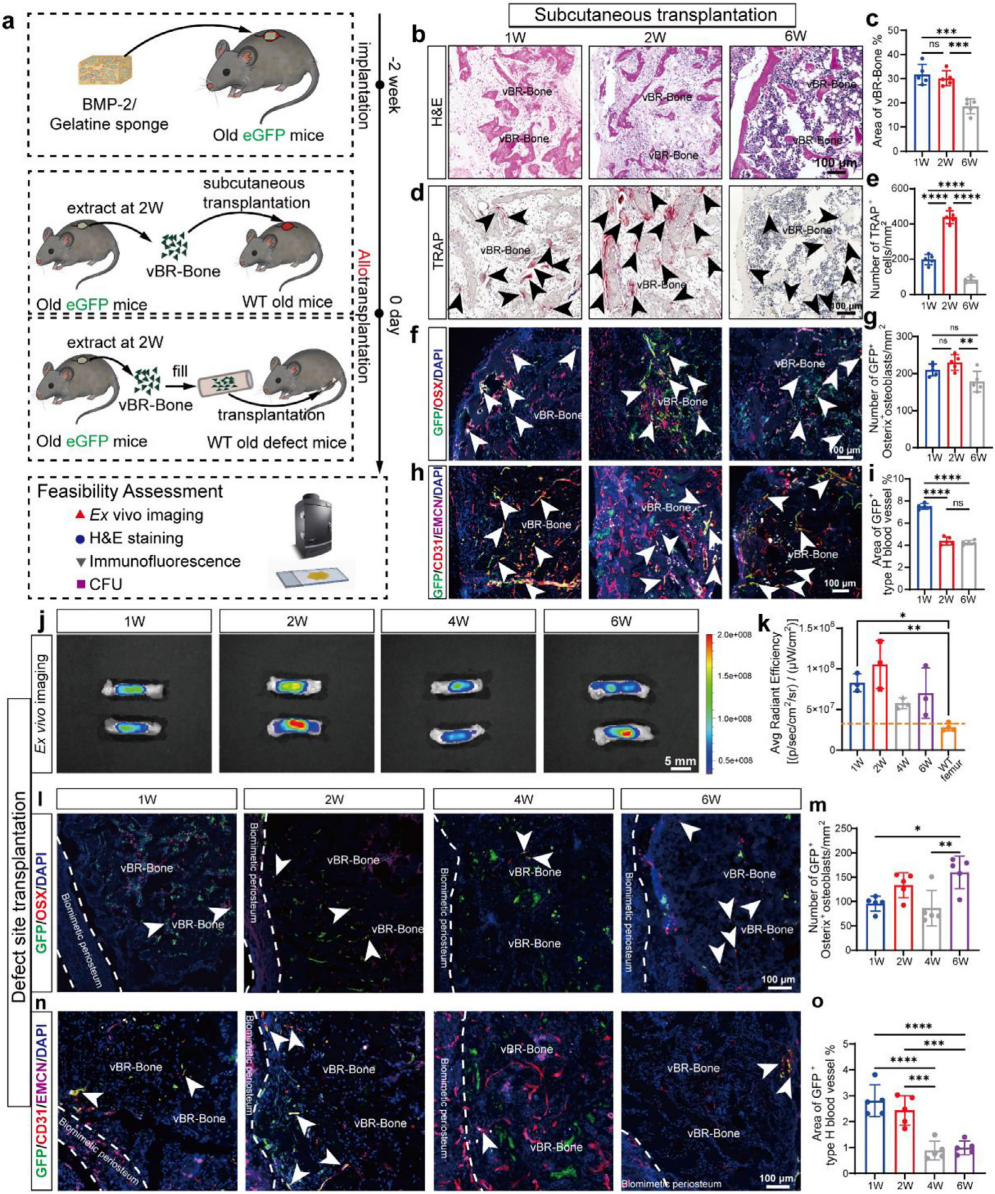
vBR‐Bone participates in osteogenesis and osseointegration during allogeneic transplantation. (a) Schematic workflow and characterization for evaluating the feasibility of allogeneic transplantation of vBR‐Bone engineered via an in vivo bioreactor in eGFP reporter aged mice. (b,c) H&E staining of vBR‐Bone at 1, 2, and 6 weeks after subcutaneous transplantation. vBR‐Bone fragments progressively remodeled into trabecular structures with newly formed marrow cavities. (d,e) TRAP staining of allogeneic subcutaneous transplantation of GFP‐labeled vBR‐Bone. (f–i) Immunofluorescence of osteoprogenitor cells and type H vessels in allogeneic subcutaneous transplantation. Black arrows: TRAP^+^ cells. White arrows: GFP^+^ osteoprogenitor cells or type H vessels. (j,k) Ex vivo imaging of allogeneic bone defect transplantation with GFP‐labeled vBR‐Bone and its quantification demonstrating vBR‐Bone survival at the defect site (N = 3). (l–o) Immunofluorescence of osteoprogenitor cells and type H vessels in allogeneic bone defect site transplantation, indicating vBR‐Bone participation in osteogenesis and osseointegration during allogeneic transplantation. White arrows: GFP^+^ osteoprogenitor cells or type H vessels. Scale bar: 100 µm (b,d,f,h,l,n), and 5 mm (j). Data are presented as the mean ± SD. ^*^ *p* < 0.05, ^**^ *p* < 0.01, ^***^ *p* < 0.001, ^****^ *p* < 0.0001, ordinary one‐way ANOVA followed by Tukey's multiple comparisons test.

To validate the feasibility of vBR‐Bone for treating critical bone defects, we established a multilayered validation system via allogeneic subcutaneous transplantation and allogeneic bone defect site transplantation assays, conducting progressive evaluations on viability and bone remodeling‐integration functions (Figure 4a). The allogeneic subcutaneous transplantation assay focused on the immunogenicity and vascularization potential of vBR‐Bone in allogeneic hosts. The vBR‐Bone from eGFP reporter aged mice was mechanically processed into fragments and transplanted into the dorsum of wild‐type (WT) aged mice. vBR‐Bone exhibited no obvious immune rejection, which was observed histologically in allogeneic hosts (Figure 4b,c) while maintaining osteoclastic activity (Figure 4d,e), which facilitated its remodeling into trabecular structures through integration. Furthermore, osteoprogenitor cells and type H vessels were abundant in vBR‐Bone. The highly vascularized network of vBR‐Bone facilitated bone remodeling (Figure 4f–i).

The allogeneic bone defect site transplantation assay further validated the survival status and bone regenerative function of vBR‐Bone in a microenvironment with concurrent mechanical loading and tissue regeneration demands. Following the clinical bone grafting protocol for critical‐sized bone defects [14], the allogeneic bone defect site transplantation assay involved filling GFP‐labeled vBR‐Bone fragments into a biomimetic periosteum to form a composite graft, which was then transplanted into the bone defect site of recipient mice. The transplanted vBR‐Bone remained in a viable state after 1, 2, 4, and 6 weeks (Figure 4j,k). Immunostaining showed gradual ingrowth of type H vessels into the biomimetic periosteum, with osteoblasts and type H vessels within vBR‐Bone participating in the repair process (Figure 4l–o). In the magnified view of bright‐field images merged with the GFP channel, donor‐derived cells from GFP‐labeled vBR‐Bone retained robust self‐renewal capacity in vitro after allogeneic transplantation into defects, although the percentage of GFP^+^ CFUs declined (Figure S5a–d).

Together, vBR‐Bone survives during allotransplantation and participates in osteogenesis and bone integration, demonstrating the feasibility of treating critical bone defects with this strategy.

**FIGURE 5:**
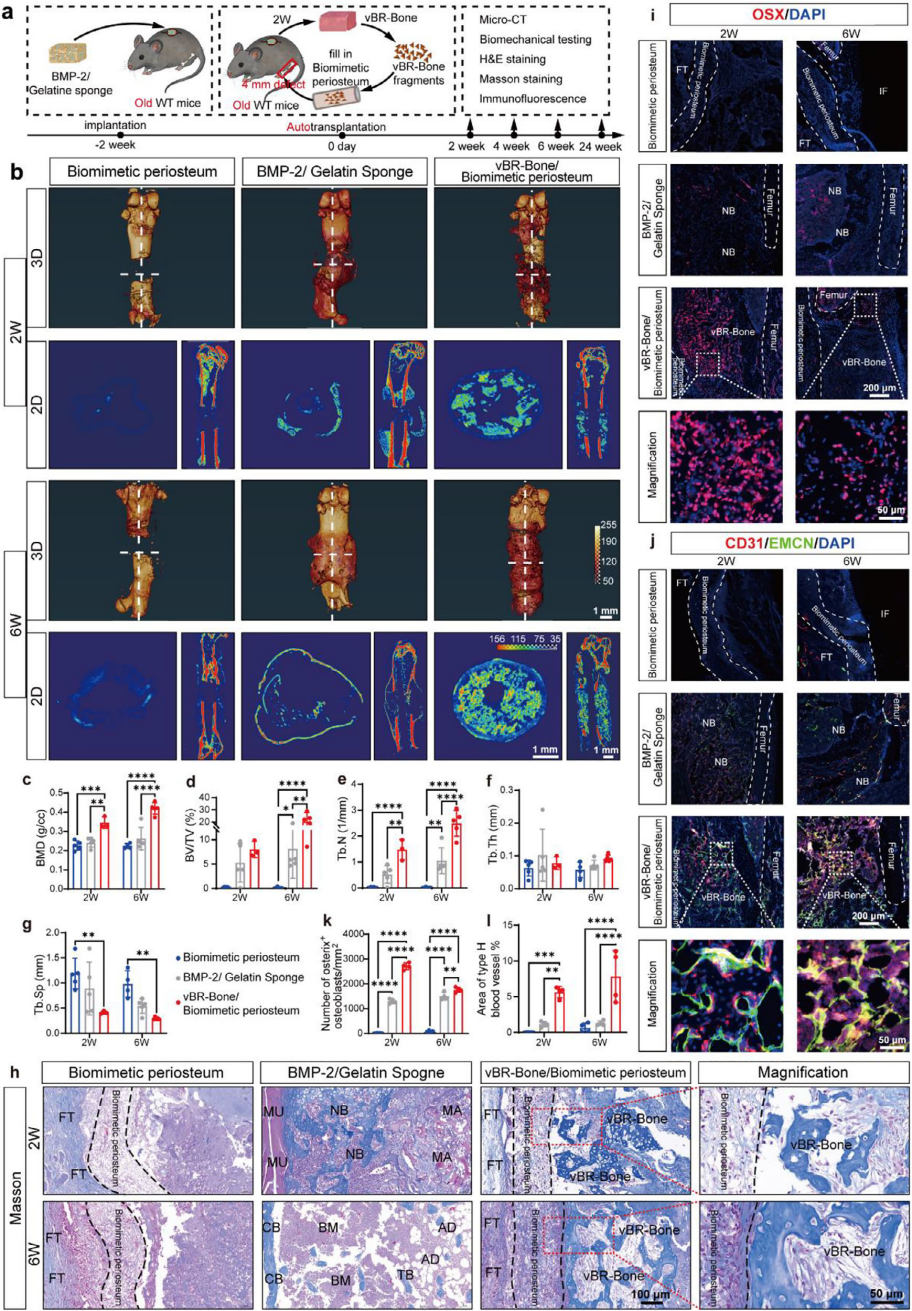
vBR‐Bone repairs critical‐sized segmental defects through a compartmentalized remodeling and integration approach. (a) Workflow and subsequent characterization of in vivo bioreactor‐engineered vBR‐Bone for treating critical bone defects. (b–g) micro‐CT 3D reconstruction images, 2D cross‐sectional views, and quantitative bone parameters of the biomimetic periosteum treatment group, BMP‐2/gelatin sponge treatment group, and vBR‐Bone/biomimetic periosteum treatment group at 2 and 6 weeks after transplantation into bone defects (N = 4–5). (h) Masson staining of critical bone defect repair in the biomimetic periosteum treatment group, BMP‐2/gelatin sponge treatment group, and vBR‐Bone/biomimetic periosteum treatment group. vBR‐Bone gradually integrates from fragments at 2 weeks into trabecular bone at 6 weeks. (i–l) Immunofluorescence staining and quantification of critical bone defect repair in the vBR‐Bone/biomimetic periosteum treatment group (N = 4). The vBR‐Bone/biomimetic periosteum group contains abundant osteoprogenitor cells and type H vessels at 2 weeks, with vessels penetrating vBR‐Bone and osteoprogenitor cells enriching around the femur at 6 weeks. FT: fibrous tissue, NB: new bone, MU: muscle, MA: material, CB: cortical bone, Tb: trabecula, AD: adipose tissue, BM: bone marrow, IF: internal fixation, vBR‐Bone: in vivo bioreactor‐derived bone. Scale bar: 1 mm (b), 200 µm (i,j), 100 µm (h), 50 µm (magnified images of h,i,j). Data are presented as the mean ± SD. ^**^ *p* < 0.01, ^***^ *p* < 0.001, ^****^ *p* < 0.0001, ordinary two‐way ANOVA followed by Tukey's multiple comparisons test.

To further evaluate the bone regenerative capacity of vBR‐Bone in treating critical bone defects, an autotransplantation experiment was conducted. A critical‐sized 4 mm segmental femoral defect model was first created in aged female mice, with the defect size representing approximately one‐third of the femoral shaft. The vBR‐bone fragment, together with the intramedullary rod used for fixation, was then placed into the biomimetic periosteum and transplanted into the defect site of the same mouse (Figure 5a; Figure S6a–f). Control groups included single biomimetic periosteum transplantation and BMP‐2/Gelatin transplantation into the defect area (Figure S6g,h).

Following vBR‐Bone/biomimetic periosteum treatment, 3D reconstruction images showed effective repair of segmental critical bone defects at 6 weeks (Figure 5b). In 2D cross‐sectional and longitudinal views, vBR‐Bone was observed to integrate from bone fragments at 2 weeks into trabecular structures at 6 weeks. In the repair process of the reconstructed femur following vBR‐Bone autologous transplantation, compartmentalized vBR‐Bone fragments were observed to progressively integrate from disaggregated vBR‐Bone segments into structured trabeculae (Figure S7a,b). Long‐term evaluation at 24 weeks post‐treatment further revealed increased bone mineral density and bone volume (Figure S7f–j), suggesting that repair occurred through a compartmentalized process where discrete vBR‐Bone fragments undergo spatially orchestrated remodeling.

In contrast, the biomimetic periosteum treatment group showed poor repair of critical bone defects, indicating that the presence of vBR‐Bone enabled effective defect repair. Compared with the BMP‐2/gelatin sponge treatment, the defect site after treatment exhibited irregular bone tissue formation; the external cortical bone was thin, while the internal tissue had low bone density with sparse trabeculae, making it prone to fractures. In contrast, at both 2 and 6 weeks, the vBR‐Bone/biomimetic periosteum treatment group exhibited higher BMD, BV/TV, trabecular number, and the smallest trabecular separation (Figure 5b–g). Subsequently, biomechanical three‐point bending tests showed that the maximum load of the vBR‐Bone/biomimetic periosteum treatment group was comparable to that of the BMP‐2/gelatin sponge group, but reached only approximately one‐quarter to one‐third of that of the native femur. We speculate that this may be attributable to the reduced regenerative capacity of aged mice and potential stress‐shielding effects associated with the intramedullary fixation model, which may decrease mechanical loading on the regenerated vBR‐Bone. Notably, the fracture deflection of the vBR‐Bone/biomimetic periosteum treatment group was higher than that of both the BMP‐2/gelatin sponge group and the native femur, indicating greater toughness. Together, these results demonstrate that vBR‐Bone efficiently reconstructs the femur via autotransplantation.

**FIGURE 6:**
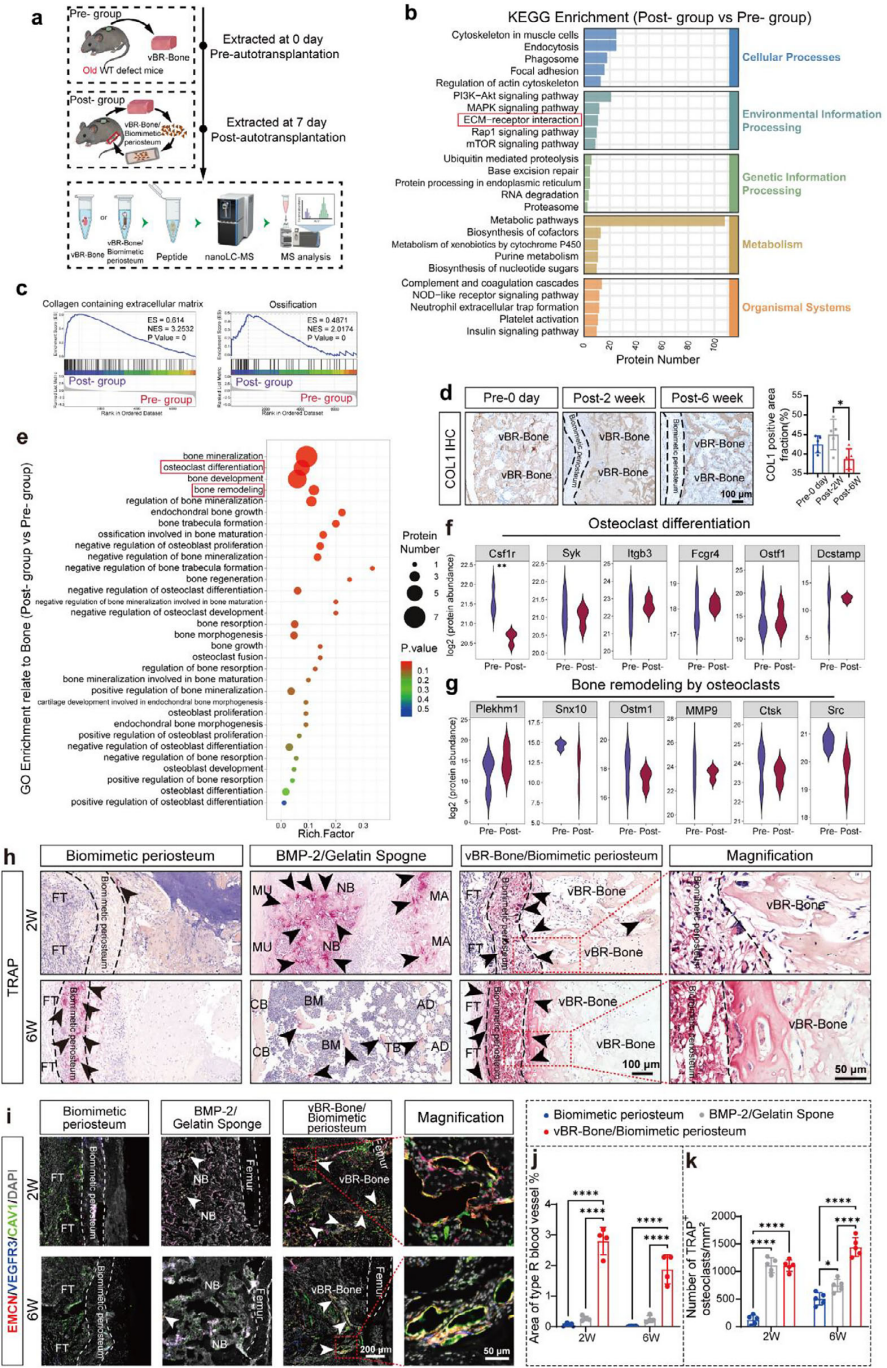
vBR‐Bone multifactors reconstitute a bone‐remodeling microenvironment at the defect site to maintain bone turnover. (a) Schematic of the animal experimental procedure for temporal dimension proteomic sample collection. Samples at 0 days pre‐autotransplantation (pre‐ group) and 7 days post‐autotransplantation (post‐ group) are shown. (b) KEGG pathway analyses comparing the post‐ group vs. pre‐ group. (c) GSEA analysis. (d) Immunohistochemical staining of COL1 and quantitative analysis of COL1‐positive area in bone tissue (N = 4). (e) GO enrichment analysis of bone‐related terms. (f,g) Violin plots depicting protein abundances associated with “Osteoclast differentiation” and “Bone remodeling by osteoclasts”. (h,k), TRAP staining and quantitative analysis of different treatment groups (N = 5). (i,j), Immunofluorescence images of type R vessels and quantitative area analysis (N = 4). FT: fibrous tissue, NB: new bone, MA: material; CB: cortical bone, vBR‐Bone: in vivo bioreactor‐derived bone. Scale bars: 200 µm (**i**), 100 µm (**d**,**h**) and 50 µm (magnified images of h,i). Data are presented as mean ± SD. ^*^ *p* < 0.05, ^**^ *p* < 0.01, ^****^ *p* < 0.0001, unpaired, two‐tailed Student's t test (f,g) and ordinary one‐way ANOVA (**d**) and two‐way ANOVA (j,k) followed by Tukey's multiple comparisons test.

Histologically, no bone tissue was formed within the biomimetic periosteum at 6 weeks. While the BMP‐2/gelatin sponge treatment group demonstrated abundant new bone formation at 2 weeks, significant cortical thinning and markedly sparse trabeculae were observed by 6 weeks, predisposing the bone to secondary fractures. In the vBR‐Bone/biomimetic periosteum group, vBR‐Bone underwent a structural evolution from fragmented segments at 2 weeks to highly organized trabeculae by 6 weeks (Figure 5h; Figure S8e). Notably, long‐term evaluation at 24 weeks revealed further maturation of the trabecular architecture, accompanied by persistent osteoclastic activity (Figure S7c—e). This process confirmed a compartmentalized regeneration approach wherein the segmental defect was partitioned into discrete microcompartments that were subsequently remodeled and integrated by vBR‐Bone fragments to reconstruct bone tissue, with CT imaging corroborating the integration and associated microarchitectural changes. Immunostaining showed that the biomimetic periosteum‐alone group lacks significant osteoprogenitor cells or type H vessels at 2 and 6 weeks during defect repair. The BMP‐2/gelatin sponge treatment group maintained relatively stable but low levels of osteoprogenitor cells and type H vessels at 2 and 6 weeks, while the vBR‐Bone/biomimetic periosteum treatment group contained abundant OSX^+^ osteoprogenitor cells and CD31^high^EMCN^high^ type H vessels within vBR‐Bone at 2 weeks, which facilitated bone integration. By 6 weeks, type H vessels were distributed throughout the vBR‐Bone, and osteoprogenitor cells were enriched in the femur and surrounding vBR‐Bone (Figure 6i–l).

Together, vBR‐Bone effectively repairs critically sized segmental defects—up to 4 mm in aged murine models, representing one‐third of the femoral shaft—through a “compartmentalized” approach that partitions the defect into manageable fragments. This approach enables vBR‐Bone fragments to serve as remodeling units that progressively reassemble into structurally integrated, functional bone tissue via autotransplantation.

### 2.5 vBR‐Bone Multifactors Reconstruct Remodeling Microenvironment

To investigate vBR‐Bone's reconstruction mechanism, proteomic analysis compared pre‐autotransplantation vBR‐Bone (pre‐group) and 7‐day post‐autotransplantation defect site tissue (post‐group) (Figure 6a). Compared to the pre‐ group, the post‐ group exhibited significant upregulation of 206 proteins and downregulation of 472 proteins (|log_2_FC | ≥ 0.263 & p < 0.05) (Figure S9a). Notably, upregulated *Loxl2* enhanced collagen cross‐linking, strengthening the extracellular matrix (ECM) (Figure S9b). GO term enrichment (top 20) revealed vBR‐Bone transplantation enhanced ECM formation and repair via ECM‐related processes (Figure S9c). These processes included “collagen‐containing extracellular matrix”, “extracellular matrix”, and “extracellular matrix organization”. KEGG analysis further identified enriched “ECM‐receptor interaction” pathways (Figure 6b), with subcellular localization predominantly detected in the extracellular region (Figure S9d). GSEA confirmed upregulation of collagen‐containing ECM, ossification (Figure 6c; Figure S9e). Upregulation of “Cell adhesion mediated by integrin” further verifies the cell‐adhesive design of biomimetic periosteum (Figure S9e). Bone comprises a matrix (primarily collagen I) and cells. Immunohistochemistry revealed abundant COL1 deposition in vBR‐Bone post‐autotransplantation, with levels exhibiting a temporal pattern that rose initially and then declined (Figure 6d), potentially linked to the processes of bone matrix remodeling and formation.

To further elucidate the mechanisms underlying vBR‐Bone incorporation into the bone matrix, we performed additional enrichment analysis on bone‐related GO terms, identifying significant enrichment of osteoclast differentiation, bone remodeling, and other bone resorption‐related terms (Figure 6e). We analyzed the expression of key factors governing osteoclast differentiation and bone remodeling by osteoclasts. While *CSF1r*, an early osteoclast differentiation factor, was decreased post‐autotransplantation, the expression levels of other differentiation markers (*Itgb3*, *Dcstamp*, *Traf6*) and remodeling enzymes (such as *MMP9*, *Ctsk*) remained comparable to pre‐autotransplantation levels (Figure 6f,g; Figure S9f). The factors associated with osteogenesis (such as *Sp7*, *Runx2*, *Postn*, *BMP‐2*, and *PDGF‐BB*) maintained or even exceeded the pre‐autotransplantation levels (Figure S9g–i). These results suggest that vBR‐Bone transplantation preserves the pre‐transplant bone remolding capacity, with multfactors in the vBR‐Bone reconstituting a bone remodeling microenvironment at the defect site.

The level of osteoclastic remodeling during critical‐sized bone defect repair was subsequently validated across the different experimental groups. TRAP staining revealed continuous osteoclastic remodeling in the vBR‐Bone/biomimetic periosteum treatment group between 2 and 6 weeks. By week 6, TRAP^+^ cells persisted peripherally near the membrane while central remodeling diminished, demonstrating near‐complete integration. In the biomimetic periosteum treatment group, intense TRAP activity localized exclusively to the biomimetic periosteum with no internal TRAP^+^ cells, suggesting preferential resorption of calcium phosphate salts. The BMP‐2/gelatin sponge group exhibited adipose tissue, sparse trabeculae, and robust osteoclastic activity at 2 weeks; by week 6, reduced TRAP^+^ cells coincided with progressive matrix absorption (Figure 6 h,k). Immunostaining revealed abundant EMCN^+^CAV^+^VEGFR3^−^ type R vessels in the vBR‐Bone/biomimetic periosteum treatment group at 2 weeks, persisting at significantly higher levels than other groups through 6 weeks despite gradual decline (Figure 6i,j). This indicated continuous active bone remodeling and maintained bone turnover after autotransplantation. As type R vessels are post‐arteriolar capillaries, their presence confirms anastomosis between the vBR‐Bone‐derived vasculature and host blood vessels, further demonstrating the viability of vBR‐Bone.

Together, these results demonstrate that the multifactors within vBR‐Bone reconstitute a bone remodeling microenvironment at the defect site, maintaining bone turnover levels.

### 2.6 vBR‐Bone‐released TGF‐β1 to Activate PI3K/AKT/mTOR via TRAF6‐Dependent Ubiquitylation for Repair

**FIGURE 7:**
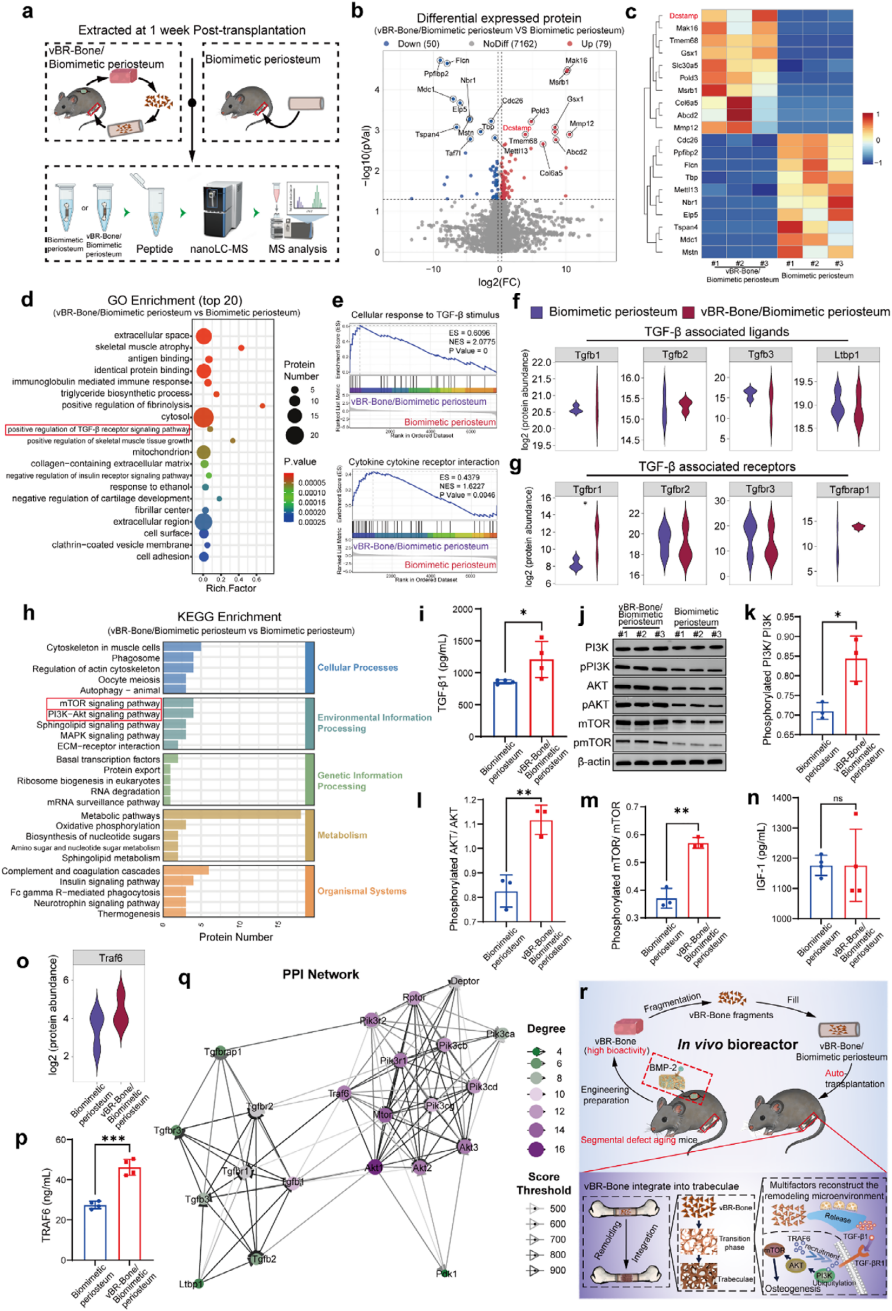
TGF‐β1 released by vBR‐Bone matrix activates PI3K/AKT/mTOR via TRAF6‐dependent ubiquitylation to promote osteogenesis. (a) Schematic of the animal experimental procedure for proteomic sample collection across different treatment groups, including the biomimetic periosteum treatment group and vBR‐Bone/biomimetic periosteum treatment group. (b,c) Volcano plot and heatmap of differentially expressed protein profiles. (d) GO enrichment analysis of the vBR‐Bone/biomimetic periosteum treatment group vs. the biomimetic periosteum treatment group. (e) GSEA analysis. (f,g) Violin plots showing the protein abundances of TGF‐β–associated ligands and receptors. (h) KEGG enrichment analysis. (i) Elisa assay for TGF‐β1 (N = 4). (j–m), Western blot of the vBR‐Bone/biomimetic periosteum and biomimetic periosteum treatment groups (N = 3). (n) Elisa assay for IGF‐1 (N = 4). (o,p) Volcano plot of TRAF6 protein abundance and elisa assay for TRAF6 (N = 4). (q) Protein–protein interaction (PPI) network associated with TGF‐β signaling pathways. (r) Schematic illustration of the workflow and mechanism of vBR‐Bone for repairing senescent segmental defects. Data are presented as the mean ± SD. ^*^ *p* < 0.05, ^**^ *p* < 0.01, ^***^ *p* < 0.001, unpaired, two‐tailed Student's t test.

Given the high osteogenic activity in vBR‐Bone, we performed proteomic analysis on femurs with defects treated for one week by the vBR‐Bone/biomimetic periosteum treatment group and the biomimetic periosteum treatment group to investigate the osteogenic role of vBR‐Bone in the bone‐remodeling microenvironment at the defect site (Figure 7a). A volcano plot of differentially expressed proteins identified 129 DEPs, with 79 upregulated and 50 downregulated proteins in the vBR‐Bone/biomimetic periosteum group compared to the biomimetic periosteum group (Figure 7b). Cluster heatmap analysis of the top 10 upregulated and 10 downregulated genes revealed that upregulated genes were associated with osteoclast development (*Dcstamp*), confirming the maintenance of remodeling capacity after vBR‐Bone transplantation (Figure 7c). GO enrichment analysis of DEGs showed significant enrichment in “positive regulation of TGF‐β receptor signaling pathway” (Figure 7d; Figure S10a). GSEA analysis demonstrated significant upregulation of biological processes involving “cellular response to TGF‐β stimulus” and “cytokine‐cytokine receptor interaction” indicating a critical role for TGF‐β in osteogenesis during bone remodeling (Figure 7e). The upregulation of both osteogenic and osteoclastic differentiation confirmed a high level of bone remodeling activity (Figure S10b). We analyzed the protein abundance of a series of TGF‐β‐associated ligands and receptors, and found that the levels of *Tgfb1*, *Tgfbr1*, and *Tgfbrap1* were significantly higher in the vBR‐Bone/biomimetic periosteum group compared to the biomimetic periosteum‐only group (Figure 7f,g).

TGF‐β1, a highly abundant bone matrix cytokine essential for bone reconstruction, is released and activated through osteoclastic resorption and matrix metalloproteinase (MMP) proteolysis [54, 55]. Consistent with the retention of elevated osteoclastic activity in vBR‐Bone transplants, we posit that osteoclastic resorption of its matrix liberates TGF‐β1. Elisa confirmed significantly higher TGF‐β1 concentrations in the vBR‐Bone/biomimetic periosteum group (Figure 7i). Given established roles of TGF‐β1 in promoting osteoblast proliferation, differentiation, and migration via PI3K/AKT/mTOR signaling [56], we performed KEGG pathway analysis. This revealed significant enrichment of DEPs in mTOR and PI3K‐Akt pathways, implicating this axis in TGF‐β1‐mediated osteogenic induction (Figure 7h). Western blotting further demonstrated enhanced phosphorylation of PI3K, AKT, and mTOR in the vBR‐Bone group, confirming pathway activation (Figure 7j‐m). While IGF‐1—another abundant bone matrix factor—can similarly activate PI3K/AKT/mTOR to drive MSC osteogenesis [57, 58], no significant IGF‐1 concentration differences were detected between vBR‐Bone/biomimetic periosteum and periosteum‐only groups (Figure 7n), excluding IGF‐1 as a primary activator in this microenvironment.

TRAF6 acts as a key mediator in TGF‐β1‐induced PI3K/AKT/mTOR activation. TGF‐β1 binding to its receptor recruits TRAF6, which catalyzes K63‐linked polyubiquitination of PI3K‐p85α (encoded by Pik3r1). This facilitates PI3K‐dependent PIP3 generation, recruiting AKT to the membrane for phosphorylation by PDK1 and mTORC2, thereby activating the pathway [59]. Furthermore, TRAF6, also a critical regulator of osteoclast differentiation, was maintained at levels consistent with its pre‐autotransplantation state of osteoclast activation (Figure S9f). In the reconstructed bone remodeling microenvironment, the abundance of TRAF6 factor was higher (Figure 7o,p), and TRAF6 was closely associated with TGF‐β receptors (*Tgfbr1*, *Tgfbr2*) and several subtypes of PI3K (*Pik3r1*, *Pik3r2*, *Pik3cd*, etc.) (Figure 7q). In correlation analysis, Tgfb1 and Tgfbr1 showed a high correlation coefficient. TRAF6 clustered with Pik3r1 (which encodes PI3K‐p85α) and was also highly correlated with Tgfbr1, demonstrating that the released TGF‐β1 activates the PI3K/AKT/mTOR axis via TRAF6 in the bone remodeling microenvironment.

Together, these results demonstrate that the multifactors within the vBR‐Bone reconstitute a functional bone‐remodeling microenvironment at the defect site. Within this niche, vBR‐Bone bone matrix‐liberated TGF‐β1 activates the PI3K/AKT pathway via TRAF6‐dependent ubiquitylation and promotes osteogenesis through a feedback mechanism via the PI3K/AKT/mTOR axis, thereby repairing critical‐sized bone defects (Figure 7r).

## 3 Discussion

In this study, we present a development‐based in vivo bioreactor strategy to treat critical segmental bone defects in aged individuals. Developmental engineering aims to cultivate organoids in vitro, but nearly all organoids face issues of insufficient vascularization [35, 39, 60]. Endothelial cells typically form only superficial capsule‐like structures, leading to hypoxic necrosis in the central region due to nutrient deprivation. Additionally, bone organoids lack maturity—either forming 3D bone‐associated cell networks without structural bone or generating mineralized bone without multicellular interactions [37, 61]. Diverging from traditional developmental engineering paradigms, the in vivo bioreactor strategy utilizes the organism itself as a bioreactor [62, 63, 64, 65]. Here, BMP‐2‐loaded biomaterials are implanted to trigger the body's innate developmental cascade. Distinct from pathological heterotopic ossification, vBR‐Bone formation follows a defined, accelerated developmental trajectory. BMP‐2 acts as a transient “on‐switch” to trigger the assembly of a self‐sustaining fibrous‐like structure typical of early osteogenesis. This in vivo bioreactor recapitulates developmental processes—including chondral ossification, bone remodeling, and marrow regeneration—generating functional structures such as trabecula, cortical bone, marrow niches, and highly organized vasculature enriched with type H and type R vessels. Moreover, our previous studies have also demonstrated that the developmental trajectory remains robust across varying dosages, where increased BMP‐2 load enhances tissue volume and density without altering the sequential progression of ossification phases [43]. These results confirm that this strategy reactivates developmental programs to engineer real bone tissue. Furthermore, our prior studies have demonstrated that this reactivated developmental process involves dynamic interactions among multiple cell types [43, 66, 67]—including hematopoietic stem/progenitor cells, immune cells, stromal cells, endothelial cells, and bone‐associated cells—confirming that this strategy faithfully recapitulates true bone tissue.

Our previous studies have enabled engineered production of functional cells for treating fibrotic diseases [43], inflammatory disorders [66], and hematopoietic injuries [43, 67, 68] by restarting developmental processes. This work focuses on controlling specific developmental stages of the in vivo bioreactor to generate highly bioactive vBR‐Bone, addressing the dual requirements for both high‐quality and large‐volume bone grafts in critical defects. Aged bone typically exhibits reduced bone turnover levels, with decreased osteoclasts and more prominently reduced osteoblasts, while vBR‐Bone shows significantly enhanced bioactivity, with elevated numbers of osteoblasts and osteoclasts. Bone regeneration‐associated type H vessels and remodeling‐associated type R vessels progressively infiltrate during development, collectively demonstrating enhanced remodeling activity. Additionally, vBR‐Bone exhibits lower senescence levels, with vBR‐MSCs displaying superior in vitro self‐renewal and osteogenic differentiation capacity compared to autologous BMSCs, confirming vBR‐Bone exhibits restoration of bone bioactivity lost during aging. This strategy enables the controllable preparation of sufficient autologous vBR‐Bone by adjusting the number of BMP‐2‐loaded biomaterials implanted, thus addressing the issues of insufficient autologous bone donor sites and poor activity of senescent autologous bone.

Clinically, autologous bone grafting typically employs morselized fragments or granules. Notably, repairing weight‐bearing femoral segmental defects remains particularly challenging due to the high mechanical and biological demands. Previous studies have shown that autologous rib fragments can integrate into functional bone structures in vivo, achieving improved bone mineral density [69, 70]. Emulating this clinical concept, we fragmented vBR‐Bone and packed the fragments into a biomimetic periosteum for transplantation. We demonstrated that vBR‐Bone fragments progressively integrate and remodel into trabecular bone, fully reconstructing the defect architecture. This reparative process aligns with a compartmentalized model in which individualized vBR‐Bone fragments serve as remodeling units that are independently incorporated into the overall bone structure. Moreover, vBR‐Bone contains abundant type H vessels and osteoprogenitor cells, which actively facilitate regeneration. Transplanted vBR‐Bone also sustained high remodeling activity, rich type R vessel presence, and elevated osteoclast counts. Since type R vessels are specialized post‐arteriolar capillaries [50], their incorporation confirms functional vascular integration between vBR‐Bone and host tissue.

Preventing fibrous tissue invasion is another critical consideration in bone defect repair [19, 25, 71]. Biological barrier membranes serve in dental/maxillofacial/cranial defects to exclude non‐osteogenic cells while selectively admitting osteogenic cells, enabling bone regeneration and augmentation [26, 72, 73, 74]. However, large segmental defects in long tubular bones require substantial bone mass for structural reconstruction, limiting their application in such defects. Periosteum is a natural barrier tissue with excellent osteogenic properties and vascular permeability [75, 76, 77]. We designed a biomimetic periosteum simulating periosteal functions to provide remodeling space for vBR‐Bone. Material selection was guided by both barrier performance and mechanobiological cues. Using clinically FDA‐approved PCL and TCP as substrates, we fabricated an asymmetric biomimetic periosteum with a hydrophilic inner layer and hydrophobic outer surface through electrospinning and plasma treatment. The electrospun architecture forms a relatively dense fibrous network that is advantageous for reducing fibrous tissue ingrowth [78]. In addition, compared with hydrogel‐based or ECM‐derived periosteum matrices, the PCL/TCP composite provides higher stiffness, which may serve as a favorable mechanobiological signal to support osteogenic differentiation [79, 80, 81]. Furthermore, allogeneic transplantation confirmed that the biomimetic periosteum permits vascular ingrowth while maintaining blood supply within the internal space.

While this study demonstrates the potential of in vivo bioreactor strategy, several limitations warrant consideration. Although we validated the “youth‐like” phenotype of vBR‐Bone using multiple approaches, the mechanistic basis remains unclear. The osteoinductive capacity of BMP‐2 is well‐established [82, 83]; however, prior studies have predominantly focused on mid‐to‐late‐stage osteogenic differentiation and mineral deposition, with less emphasis on early developmental events. Notably, we found that BMP‐2 stimulation can induce the formation of a fibrous‐like structure characteristic of early bone formation, which histologically mirrors the initiation phase of skeletal development. Therefore, we postulate that this “youth‐like” phenotype may stem from the reactivation of a developmental program, effectively reverting cells and tissues to a relatively earlier, more primitive state. Regarding the spatial control of this potent inductive signal, we speculate that the surrounding fibrous capsule formed during the early bone formation functions as a physical barrier to confine the process, thereby preventing off‐target BMP‐2 diffusion and ectopic muscle ossification. Additionally, this phenomenon may involve epigenetic regulation, a hypothesis that requires further validation in future studies.

Beyond these mechanistic considerations, additional limitations related to translational relevance should be acknowledged. Findings derived primarily from aged murine models require validation in large animals and ultimately human patients for translational relevance, given inherent interspecies differences in bone metabolism, immune responses, and lifespan. Furthermore, the cellular origins and lineage dynamics within vBR‐Bone remain incompletely characterized, necessitating definitive lineage tracing to elucidate host cell interactions during remodeling. Additionally, the long‐term biocompatibility and degradation kinetics of the biomimetic periosteum require further investigation to assess risks of chronic foreign body responses. Finally, scalability and standardization hurdles for clinical translation—including protocols for implantation, harvesting, and quality control—must be addressed.

Advancing this strategy toward clinical application necessitates sequential steps. Validation in large animal models is critical to assess scalability and mechanical performance in critical weight‐bearing defects. While standardized protocols have been established in mouse models [62], they remain unexplored for larger animals. Concurrently, developing a standardized real‐time monitoring system for visualizing vBR‐Bone development stages is clinically imperative to define optimal harvest windows and guide subsequent surgical procedures. Mechanistically, definitive lineage tracing studies remain essential to elucidate donor‐host cell dynamics and persistence thresholds governing remodeling. Although the cellular origins within the in vivo bioreactor await full resolution, this approach provides a robust platform for harvesting abundant, high‐activity autologous bone tissue. Successful implementation of this strategy will ultimately deliver a transformative therapeutic paradigm for elderly patients with severe weight‐bearing bone defects.

## 4 Conclusion

Inspired by developmental principles, this study presents an innovative in vivo bioreactor strategy using BMP‐2‐loaded biomaterials to activate endogenous bone developmental programs. The resulting tissue—termed in vivo bioreactor‐derived bone (vBR‐Bone)—faithfully recapitulates native osseous architecture, including cortical and trabecular bone, functional marrow niches, and hierarchically organized vasculature. vBR‐Bone exhibits rejuvenated restoration of bone bioactivity lost in aging, marked by reduced senescence, elevated remodeling dynamics, and enhanced stem cell functionality. When encapsulated in an asymmetric biomimetic periosteum that spatially blocks fibrous tissue invasion while permitting vascular ingrowth, vBR‐Bone fragments repair critical‐sized segmental defects in aged mice via a “compartmentalized” remodeling‐integration process. Mechanistically, vBR‐Bone‐derived multifactors reestablish a bone‐remodeling microenvironment in which matrix‐liberated TGF‐β1 activated the PI3K/AKT/mTOR signaling axis through TRAF6‐dependent ubiquitylation, driving vigorous osteogenesis. Collectively, this developmental in vivo bioreactor strategy overcomes limitations of autograft scarcity and age‐related regenerative decline, offering a clinically viable and biologically grounded solution for reconstructing challenging bone defects.

## 5 Materials and Methods

### 5.1 Preparation of BMP‐2‐loaded Biomaterials

Based on previously reported methods, BMP‐2‐loaded biomaterials were prepared [62]. In brief, under sterile conditions, 10 µL of recombinant human bone morphogenetic protein‐2 solution (concentration of 1 mg/mL, Shanghai Rebone Biomaterials Co., Ltd.) was added to a porous absorbable gelatin sponge (length 5 mm, thickness 5 mm, height 5 mm, Jiangxi Xiangen Co., Ltd.). Then, the gelatin sponge scaffold loaded with BMP‐2 was freeze‐dried to obtain bioactive materials, which were stored at −20°C for future use.

### 5.2 Construction of an In Vivo Bioreactor

Wild‐type C57BL/6 female mice aged 16 months used in this study were purchased from Shanghai Shengchang Company. Firstly, gas anesthesia with isoflurane was used to anesthetize the mice. The back hair was shaved, and the skin on the back of the mice was incised using surgical scissors. A BMP‐2‐loaded gelatin scaffold was subcutaneously implanted and underwent bone formation in in vivo bioreactor. The site of this developmental process is termed an in vivo bioreactor. The engineered tissue serves as the vBR‐Bone.

### 5.3 Histological Staining

Freshly isolated natural bone (femur) and engineered vBR‐Bone were fixed in 4% paraformaldehyde for 24 h, followed by decalcification in 0.5 m ethylenediaminetetraacetic acid (EDTA) for one week. The decalcified tissues were placed in embedding cassettes, dehydrated in a gradient, and then infiltrated with paraffin. After the paraffin solidified, the wax blocks were removed from the embedding molds and trimmed. The embedded wax blocks were sectioned into 10 µm thick slices using a microtome, and the slices were mounted on glass slides. The paraffin sections were deparaffinized in a gradient and stained using the corresponding staining kits, including hematoxylin and eosin (H&E), Saf‐O/fast green staining, and tartrate‐resistant acid phosphatase (TRAP).

For the repair of segmental femoral defects at two and four weeks, the femurs were fixed for 24 h, decalcified for one week, and then embedded in paraffin. The paraffin sections were stained using the corresponding staining kits, including hematoxylin and eosin (H&E), Masson's trichrome, and tartrate‐resistant acid phosphatase (TRAP) staining.

### 5.4 Micro CT

Micro‐computed tomography (µCT) imaging was performed using a high‐resolution µCT scanner (Skyscan1272, Bruker, USA) to assess the development of vBR‐Bone and the repair of critical‐sized femoral defects. The scanner was set at 60 kV and 160 µA, with a pixel resolution of 6.53415 µm. 3D reconstruction was conducted using Avizo 2020.1 software, with a grayscale range set from 50 to 255 to differentiate bone tissues of different densities. The reconstructed slices were color‐coded using the physics.icol colormap, with a range of 35‐156. CTAn software (Bruker microCT, Kontich, Belgium) was used to calculate bone volume/total volume (BV/TV), bone mineral density (BMD), trabecular number, trabecular thickness, and trabecular separation.

### 5.5 Animal Experiments

All the female C57BL/6 mice, aged 16 months, were purchased from Shanghai Shengchang Co., Ltd. All the eGFP mice, aged 16 months, were purchased from Cyagen Biosciences Inc. The mice were housed in the Laboratory Animal Center of Shanghai Jiao Tong University. All experimental procedures were approved by the Institutional Animal Care and Use Committees of Shanghai Jiao Tong University (A2023198).

This study established a 4 mm critical‐size femoral defect model in mice. Female C57BL/6 mice (16 months old) were randomly assigned to three groups (N = 4–5 biological replicates per group at each time point) for femoral defect construction. The groups included the biomimetic periosteum treatment group (biomimetic periosteum transplantation only), the BMP‐2/Gelatin treatment group (transplantation of BMP‐2/Gelatin), and the vBR‐Bone/biomimetic periosteum treatment group (obtaining pre‐constructed vBR‐Bone, processing them into bone fragments, and filling them into the biomimetic periosteum for composite transplantation into the defect). Briefly, under isoflurane anesthesia, a 10 mm incision was made on the skin over the femoral shaft of the left hind leg. Muscles were dissected to expose the femoral shaft, and a 4 mm defect was created using a circular saw. Stainless steel rods (0.8 mm diameter, 11 mm length) were inserted into the medullary cavity on both sides for internal fixation. Different graft materials were implanted into the defects, followed by wound closure using sutures (Jinhuan Medical). In the vBR‐Bone/biomimetic periosteum treatment group, prior to transplantation, an in vivo bone reactor was constructed subcutaneously on the back of the mice two weeks in advance. vBR‐Bone was obtained at the time of treatment, mechanically processed into fragments, and filled into the biomimetic periosteum before transplantation into the defect.

For allotransplantation, eGFP reporter female mice (16 months old) were used as donors (N = 6 biological donors), and C57BL/6 female mice (16 months old) were used as recipients (total N = 24 biological recipients). The vBR‐Bone constructed subcutaneously on the back of eGFP reporter donor mice were harvested and mechanically processed into fragments. For subcutaneous allotransplantation, eGFP‐expressing vBR‐Bone fragments were transplanted into a subcutaneous fascial pouch at an anatomically distinct site in the same recipient mouse to allow re‐integration (N = 4 biological recipients per time point). Recipients were euthanized at 1, 2, and 6 weeks postoperatively for characterization and analyses. For the femoral defect allotransplantation experiment, eGFP‐expressing vBR‐Bone fragments were loaded into the biomimetic periosteum and transplanted into the femoral defect in C57BL/6 recipient female mice using the same femoral defect model and surgical procedures described above (N = 3 biological recipients per time point). Recipients were euthanized at 1, 2, 4, and 6 weeks postoperatively for characterization and analyses.

### 5.6 Statistical Analysis

Statistical analysis was conducted using GraphPad Prism version 9.0.0.121 (GraphPad Software, San Diego, CA, USA). Data are presented as mean ± SD. For comparisons between two groups, an unpaired, two‐tailed Student's t‐test was used. For comparisons among three or more groups, one‐way or two‐way analysis of variance (ANOVA) was applied as appropriate, followed by Tukey's post‐hoc multiple‐comparisons test. Statistical significance was defined as follows: ^*^ *p* < 0.05, ^**^ *p* < 0.01, ^***^ *p* < 0.001, and ^****^ *p* < 0.0001.

## Author Contributions

Conceptualization: WCZ, KD, JW; Methodology: WCZ, TS, KD, ZHG, FYY, XLW, YKF, SZZ; Investigation: WCZ, KD Visualization: WCZ; Funding Acquisition: KD, JW, CSL Project Administration: JW, CSL; Supervision: JW, CSL Writing – Original Draft: WCZ, KD; Writing – Review & Editing: WCZ, KD, JW, CSL.

## Conflicts of Interest

The authors declare no conflicts of interest.

## Supporting information




**Supporting File**: advs74727‐sup‐0001‐SuppMat.docx.

## Data Availability

The data that support the findings of this study are available from the corresponding author upon reasonable request.
