# Supplementary material for: Development‐based In Vivo Bioreactor Strategy for Challenging Senescent Bone Reconstruction
Source: Adv Sci (Weinh). 2026 Mar 9;13(28):e22408. doi: 10.1002/advs.202522408 (PMC13185884; doi:10.1002/advs.202522408)
Supplement: Supplementary file 1 — Supporting File: advs74727‐sup‐0001‐SuppMat.docx. [file ADVS-13-e22408-s001.docx]

**List of SUPPLEMENTAL INFORMATION:**

Materials and Methods

Figure S1-10

Table S1

**Materials and Methods**

**Immunofluorescence and immunohistochemistry**

For immunofluorescence staining, decalcified samples were soaked in a 20% sucrose solution for 48 hours to dehydrate them. Then, the specimens were embedded in OCT medium and sliced to a thickness of 10 μm or 40 μm using a cryostat (at -20 ℃). The frozen sections were left at room temperature for 30 minutes and rehydrated with ultrapure water. They were rinsed twice with TBST for 5 minutes each, followed by permeabilization with 0.3% Triton × 100 in TBST for 20 minutes (prepared by diluting 3% Triton × 100 tenfold in TBST). Blocking was performed with 5% (v/v) donkey serum (dissolved in 0.1% Triton × 100) at room temperature for 1 hour (diluted thirtyfold with 3% Triton). After blocking, the sections were incubated with primary antibodies overnight at 4 °C. The primary antibodies used were rabbit anti-Osterix (Abcam, 1:200), goat anti-Leptin receptor biotinylated antibody (R&D, 1:100), rabbit anti-CD31 (Abcam, 1:100), rat anti-EMCN (Santa Cruz Biotechnology, 1:100), rabbit anti-CTSK (Proteintech, 1:100), goat anti-VEGFR3 (R&D, 1:50) and rabbit anti-Cav1 (CST, 1:50 dilution). The next day, after washing off the primary antibodies with TBST, appropriate fluorescently labeled secondary antibodies were applied for 1 hour. The secondary antibodies used were donkey anti-goat Alexa Fluor 488 (1:400), donkey anti-rabbit Alexa Fluor 594 (1:400), donkey anti-rat Alexa Fluor 488 (1:400), donkey anti-rabbit Alexa Fluor 647 (1:400), donkey anti-rat Alexa Fluor 594 (1:400), and donkey anti-goat Alexa Fluor 647 (1:400). The sections were mounted with Prolong Gold antifade reagent containing DAPI (CST). The fixed sections were imaged using a laser scanning confocal microscope (Leica SP8).

**Senescence-associated β-galactosidase (SA-β-gal) staining**

For Sa-β-gal staining of bone tissue sections, the decalcified samples were replaced with a solution of 20% (w/v) sucrose and 2% polyvinylpyrrolidone (PVP, Sigma-Aldrich) in PBS at 4 ℃ to remove water. The samples were transferred to molds, frozen, and stored at -80 ℃ until sectioning. Frozen decalcified samples were sectioned to a thickness of 10 μm using a cryostat with a low-temperature chamber set at 20 ℃ and air-dried. After rehydration in PBS, staining was performed using a cellular senescence β-galactosidase staining kit (Beyotime).

Frozen sections were first thawed, and the tissue was washed three times with PBS, each time for no less than 5 minutes. An appropriate volume of β-galactosidase staining fixative was added for fixation at room temperature for 30 minutes. After three washes with PBS, an appropriate amount of staining working solution was added. The samples were incubated overnight at 37 ℃ (except in a CO_2_ incubator) and can be covered with parafilm or plastic wrap to prevent evaporation. Observation was done under a conventional light microscope.

For Sa-β-gal staining of cells, wash with PBS and add an appropriate amount of β-galactosidase staining fixative for fixation at room temperature for 15 minutes. Wash the cells with PBS and add 1 mL of staining working solution per well. Incubate overnight as described above and image.

**Cell culture and cell experiments**

BMSCs and vBR-MSCs were extracted following previous methods^65^. The complete culture medium was prepared by adding 10% fetal bovine serum, 1% penicillin-streptomycin (10,000 U/mL; Gibco), 1% sodium pyruvate (100 mM; Gibco), and 0.1% Rho-associated kinase inhibitor (Y-27632 dihydrochloride; 10 mM dimethyl sulfoxide, 1 mL) to MEM-α medium. The cell suspension was seeded in 6-well plates or 100 mm cell culture dishes containing complete culture medium and incubated at 37 °C in a 5% O_2_, 5% CO_2_ incubator.

**CFU-F assay**

For CFU-F detection, MSCs were directly cultured in 6-well plates with complete culture medium (DMEM supplemented with 10% fetal bovine serum, 1% penicillin-streptomycin (10,000 U/mL; Gibco), 1% sodium pyruvate (100 mM; Gibco), and 0.1% Rho-associated kinase inhibitor (Y-27632 dihydrochloride; 10 mM dimethyl sulfoxide, 1 mL)) for 10 days. After washing with PBS, the MSCs were fixed in 4% (w/v) paraformaldehyde for 10 minutes and stained with crystal violet staining solution. After rinsing with ddH_2_O, colony formation was observed under an optical microscope (DMi8; Leica). Macroscopic images of the wells were captured using a camera. GFP-labeled CFUs were imaged using an APX100 imaging system (Evident).

**Osteogenic differentiation experiment**

The osteogenic differentiation of MSCs from different sources was evaluated through ALP staining and ARS staining. In brief, MSCs were seeded in 24-well plates (2 × 10^4^ cells per well) and cultured. When the cell density reached 70%-80% in the wells, complete MEM-α medium was replaced with osteogenic induction medium. The osteogenic induction medium consisted of complete DMEM supplemented with 0.1 × 10^-6^ M dexamethasone (Sigma), 50 × 10^-6^ M ascorbic acid (Sigma), and 10 × 10^-3^ M β-glycerophosphate (Sigma). The osteogenic induction medium was changed every 48 hours.

For ALP staining, after 7 days, samples were stained using the BCIP/NTB alkaline phosphatase chromogenic kit (Beyotime) and visualized under an optical microscope (Axioskop 2 plus, ZEISS, Germany).

The ARS assay was used to evaluate the mineral deposition of MSCs in osteogenic induction medium. On day 14, the samples were fixed in 4% paraformaldehyde for 10 minutes after being washed with PBS, followed by staining with the ARS staining kit (Beyotime). After a 10-minute incubation, the excess dye was removed by washing with PBS. The stained samples were then observed under an optical microscope. To dissolve the dye on the samples, they were incubated in 10% cetylpyridinium chloride (CPC, Sigma Aldrich, USA). The absorbance was measured using a spectrophotometer at 540 nm for quantitative analysis.

**Real-time RT‒PCR analysis**

Total RNA was prepared using TRIzol (Takara) and reverse transcribed into cDNA with the PrimeScript RT Reagent Kit (Takara, PR047A). Real-time reverse transcriptase RT-PCR was performed with the Bio-Rad CFX96 system. The qPCR primers used are shown in Table S1.

**The preparation of an asymmetric double-layer biomimetic periosteum**

PCL was dissolved in a mixed organic solution consisting of 75% (v/v) THF and 25% (v/v) DMF at a concentration of 15% (wt/v). Various concentrations of TCP (0% (wt/v), 3% (wt/v), and 10% (wt/v)) were added to prepare spinning solutions, which were stirred at room temperature for 24 hours. Subsequently, the spinning solution underwent a 5-minute ultrasonic treatment prior to electrospinning. Each spinning solution was dispensed into a 20 mL plastic syringe equipped with a blunt-tipped needle with a 25 G size and 0.25 mm inner diameter. The spinning solution was then injected into the needle tip at a rate of 0.045 mm/min. The applied voltage was set at 10 kV, with the spinning chamber temperature maintained at 40 °C and the humidity at 20%. To act as the collector, a stainless-steel rod with a diameter of 2.5 mm was placed 15 cm away from the needle tip. Under the influence of the applied high voltage, the spinning solution formed fibers in a Taylor cone shape at the needle tip and deposited onto the rotating metal rod connected to the negative electrode, with a rotation speed of 100 rpm.

Electrospinning was initiated by placing the collector rod in a receiving position for 10 minutes, resulting in the formation of the inner layer of biomimetic periosteum. Subsequently, the metal rod, along with the electrospinning membrane, was introduced into a plasma processing machine equipped with a plasma cleaner for oxygen plasma treatment. The electrospun PCL membrane was carefully positioned inside the plasma cleaner chamber, and a radiofrequency power of 70 W was applied. Operating in a vacuum mode, pure oxygen was slowly introduced into the chamber until the pressure reached 0.4 mbar. The plasma discharge lasted for 1 minute. The metal rod, now carrying the treated electrospinning membrane, underwent an additional spinning process for 5 minutes to fabricate a dual-layer biomimetic periosteum with amphiphilic properties. Afterwards, the biomimetic periosteum was placed in a constant temperature oven set at 37 °C and left overnight to ensure complete drying. The following day, it was removed from the metal rod and utilized for experimental purposes.

**Characterization of the physicochemical properties of the asymmetric biomimetic periosteum**

The morphology of the two sides and cross-section of the samples were characterized using SEM. The longest side of irregular pores in the samples was measured and analyzed using Image J software. The elemental composition of the samples was detected using EDS. The hydrophilicity of the inner and outer layers of the membrane was evaluated using a contact angle tester. The tensile properties of the membrane were assessed using a universal tensile testing machine.

**The biocompatibility of the biomimetic periosteum**

Cell adhesion behavior was assessed by staining the cellular cytoskeleton with phalloidin-FITC, imaging under a confocal microscope, and evaluating cell imaging using environmental scanning electron microscopy.

*In vitro* cell cytotoxicity experiment. According to the national standard GB/T14233.2-20XX, an *in vitro* cell cytotoxicity test was conducted by incubating cells with the extraction medium from the biomimetic periosteum. Each sample was cut into segments of 1 cm in length and subjected to 12 hours of UV irradiation for sterilization. Under sterile conditions, the samples were incubated in a 3 cm^2^/mL mixture of MEM-α containing 10% fetal bovine serum and 1% antibiotics (penicillin and streptomycin) at 37 °C and 5% CO_2_ for 72 hours to obtain the extraction medium. After sterilization with a 0.22 μm microporous membrane, human mesenchymal stem cells (MSCs) were seeded in a 96-well plate at a cell density of 1000 cells/well and allowed to attach for 8 hours. The original culture medium was removed, and 200 μL of the extraction medium was added and continued to incubate. The control group was treated with MEM-α containing 10% fetal bovine serum and 1% antibiotics. After 1, 3, and 7 days of medium replacement, a CCK-8 cell toxicity assay was performed according to the instructions of the kit. In summary, after adding 20 μL of CCK-8 solution to each well, the plate was incubated for 1 hour in a cell culture incubator. After incubation, the optical density (OD) was measured at a wavelength of 450 nm using an ELISA reader. The difference in OD values between the experimental and control groups was used to determine cell cytotoxicity.

*In vivo* biocompatibility experiment. The biomimetic periosteum was cut into segments of 1 cm in length, opened along the incision line, and then sterilized under UV irradiation for 12 hours. Subcutaneous implantation was performed on the back of mice, with the hydrophilic side of the membrane facing the inner muscle and the other side in contact with the outer skin. Samples were collected after 1, 3, and 7 days for evaluation of *in vivo* biocompatibility using H&E staining.

**The induction of osteogenesis experiment using the biomimetic periosteum**

ALP staining was used to evaluate the osteogenic induction ability. The sterilized biomimetic periosteum was co-cultured with MSCs in 24-well plates (2 × 10^4^ cells per well). When the cell confluence reached 70%, the complete culture medium was replaced with osteogenic induction medium, consisting of complete MEM-α supplemented with 0.1 × 10^-6^ M dexamethasone, 50 × 10^-6^ M ascorbic acid, and 10 × 10^-3^ M β-glycerophosphate. The osteogenic induction medium was replaced every 48 hours. After 7 days of induction, ALP staining was performed using an ALP staining kit. The ALP activity was quantitatively evaluated using an alkaline phosphatase assay kit.

**Bio-mechanical analysis**

The three-point bending test was conducted to evaluate the biomechanical properties of the femur after treatment. After 6 weeks of treatment for the biomimetic periosteum group, BMP-2/Gelatin group, and vBR-Bone/biomimetic periosteum group in the femoral defect model, the treated femurs were fixed in 4% paraformaldehyde for 24 hours and stored at -80 °C. On the day of analysis, the femurs were thawed at room temperature, and excess moisture was removed with lint-free paper until the biomechanical testing was performed. The experiment was performed using a three-point bending fixture attached to the Miniature Electromagnetic Multiaxial Dynamic Mechanical Testing System (BTC-E-006), with a load cell of 30 N. Prior to formal testing, the instrument was calibrated, and the wall thickness of the femur's major and minor axes was measured using a caliper. The femur was placed on the machine fixture, and the span was adjusted to 7 mm. The instrument probe was adjusted to make contact with the femur, and the load sensor was zeroed at this point. The probe was then lowered at a speed of 0.05 mm/s until the femur fractured. The fracture displacement and fracture load were obtained from the automatically recorded “displacement-load” curve.

***Ex vivo* imaging**

The transplanted vBR-Bone from eGFP old C57 female mice to WT mice with femoral defects was undergo *ex vivo* imaging at specified time points using a small animal living optical imaging system (PerkinElmer IVIS Lumina III). The fluorescence intensity was analyzed using Living Image software, with the color scale set from 3.4×10^7^ to 2.0×10^8^.

**Western blot**

Tissue blocks from the 7-day femoral defect areas of the vBR-Bone/biomimetic periosteum treatment group and biomimetic periosteum treatment group were sampled, washed 2-3 times with pre-cooled PBS to remove blood contaminants, cut into small pieces, and placed into a homogenization tube. Two 4-mm homogenization beads and 10 times the tissue volume of lysis buffer (Cell lysis buffer for Western and IP, Beyotime) were added for homogenization. After homogenization, the sample was centrifuged at 12,000 rpm for 10 min at 4 °C, and the supernatant was collected as the total protein solution. Protein concentration was quantified using a BCA kit (Beyotime). Equal amounts of protein were separated from each sample using BeyoGel™ Plus PAGE SDS-PAGE gel (PO508M) and then transferred to a PVDF membrane (Millipore). After blocking with blocking buffer (Beyotime, P0252) at room temperature for 30 min, the Western blot was incubated overnight at 4°C with rabbit primary antibodies, including β-Actin (Cell Signaling Technology, 4967S), PI3K (STARTER, S0B0265), phosphorylated PI3K (Cell Signaling Technology, 4228T), AKT (Servicebio, GB15689), phosphorylated AKT (Cell Signaling Technology, 9275T), mTOR (Affinity, AF6308), and pmTOR (Servicebio, GB114489). HRP-conjugated goat anti-rabbit antibody (Cell Signaling Technology, 7074) was used as the secondary antibody to detect target proteins. Visual analysis was performed using a chemiluminescent imaging system (Tanon, Shanghai, China).

**ELISA analysis**

We analyzed TGF-β1 ELISA (NeoBioscience, EMC107b.48), IGF-1 ELISA (NeoBioscience, EMC125.48), PDGF-BB ELISA (NeoBioscience, EMC032.48), TRAF6 (ELK Biotechnology, ELK10900) and BMP-2 ELISA (NeoBioscience, EHC172.96) by using a kit. All ELISA assays were run according to the manufacturer’s instructions

**Proteomics analysis**

Proteomics analysis was performed on vBR-Bone samples collected at 0 day before autotransplantation (defined as the Pre- group) and the repaired femoral defect area at 7 days after vBR-Bone/biomimetic periosteum autotransplantation (Post- group) for time-dimensional analysis, as well as on the 7-day femoral defect areas of the vBR-Bone/biomimetic periosteum treatment group and biomimetic periosteum treatment group for intergroup comparison (N = 3). For proteomics analysis, samples were processed as follows: tissues were lysed with 8M UREA/50 mM Tris-HCL containing 1X Roche Cocktail, disrupted by grinder (60 HZ, 2 min), centrifuged, and supernatant was treated with DTT (10mM, 37 ℃ 1h) and IAA (20 mM, dark 30 min). Protein quantification via Bradford method used BSA standards to generate OD595-based standard curve, with SDS-PAGE (10 μg protein, 4-12% gel, 80 V/ 20min + 120 V/ 60min) for quality control. For digestion, 150 μg protein was digested with Trypsin (50:1 ratio, 37 ℃ 14-16 h), peptides were desalted, vacuum-dried, and reconstituted. Mass spectrometry (Nano-LC-MS/MS) used Thermo Vanquish UHPLC with 24-min gradient elution (mobile phases A/B) and Thermo Astral mass spectrometer in DIA mode (normalized collision energy 25%, resolution 240,000). Data were analyzed by DIA-NN software (version 1.9) with DirectDIA mode, Trypsin/P digestion, fixed modification Carbamidomethyl(C), and Target-decoy strategy (PSM FDR<0.01, protein FDR<0.01). Bioinformatic analysis was performed using the OmicStudio tools from LC-BIO Co. Ltd. (Hangzhou, China)

# Figure S1-10


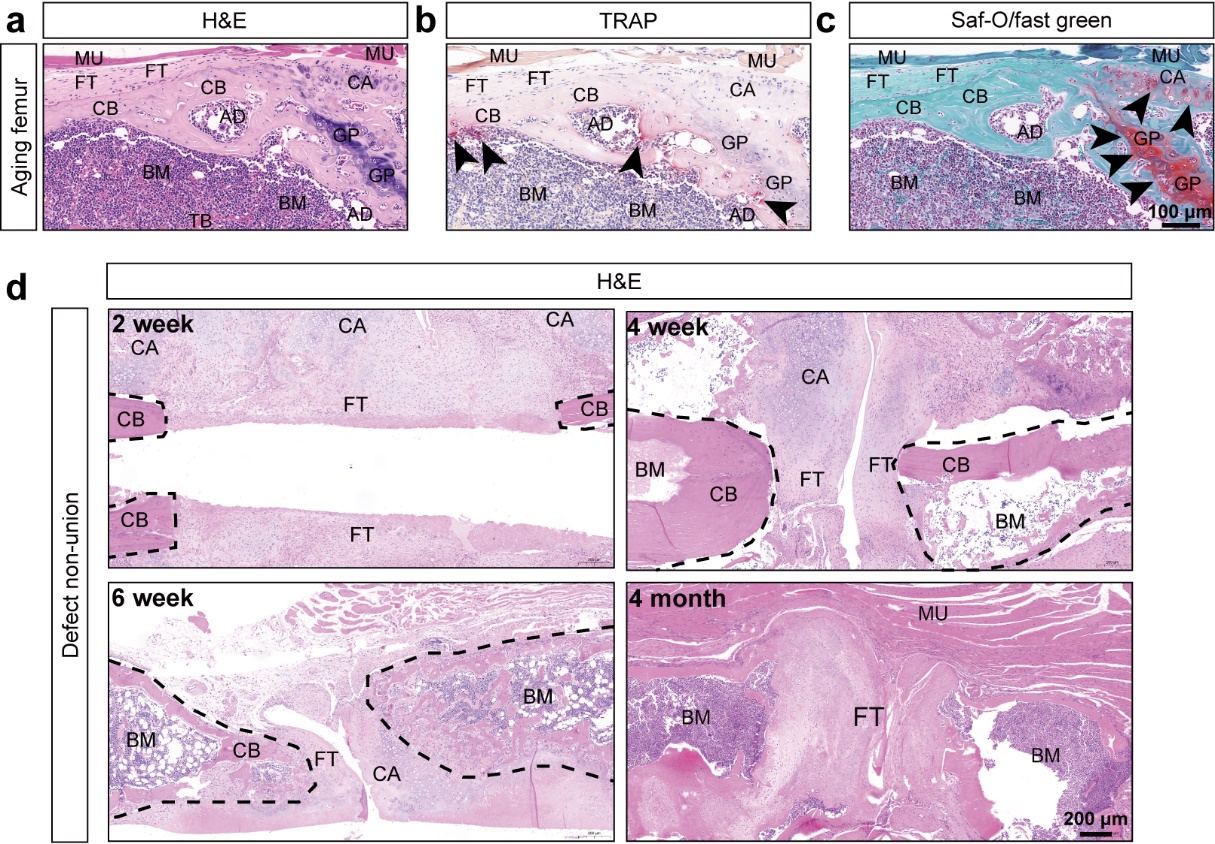


**Figure S1. Histological staining of femurs from aged mice. a**, H&E staining of aging femur. **b**, TRAP staining. **c**, Safranin O-fast green staining. **d**, H&E staining of non-union formed after bone defect. GP: growth plate, FT: fibrous tissue, CA: cartilage, MU: muscle, CB: cortical bone, Tb: trabecula, AD: adipocytes, BM: bone marrow. Scale bar: 100 μm (**a**-**c**), 200 μm (**d**).


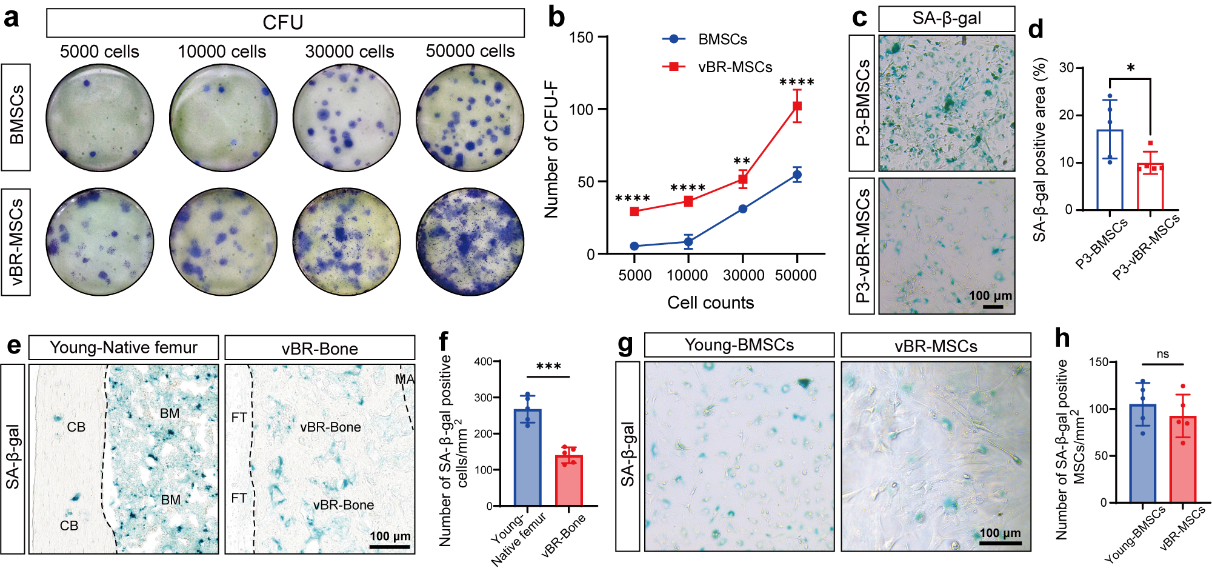


**Figure S2.** ***In vitro* self-renewal capacity and senescence characterization of MSCs.** **a**,**b**, Colony-forming unit (CFU) numbers of vBR-Bone-derived MSCs (vBR-MSCs) at different concentrations vs. bone marrow-derived MSCs (BMSCs). **c**,**d**, SA-β-gal staining of passage 3 MSCs derived from vBR-Bone in aged mice and BMSCs from aged femurs. **e**,**f**, SA-β-gal staining and quantification of femurs from young mice (6-8 weeks) and vBR-Bone generated in aged mice (16 months). CB: cortical bone, BM: bone marrow, FT: fibrous tissue, MA: material. **g**,**h**, SA-β-gal staining and quantification of BMSCs isolated from young mice (Young-BMSCs) and vBR-MSCs. Scale bar: 100 μm. Data are presented as the mean ± SD. *P < 0.05, **P < 0.01, ***P < 0.001, ****P < 0.0001, Unpaired, two-tailed Student’s t test.


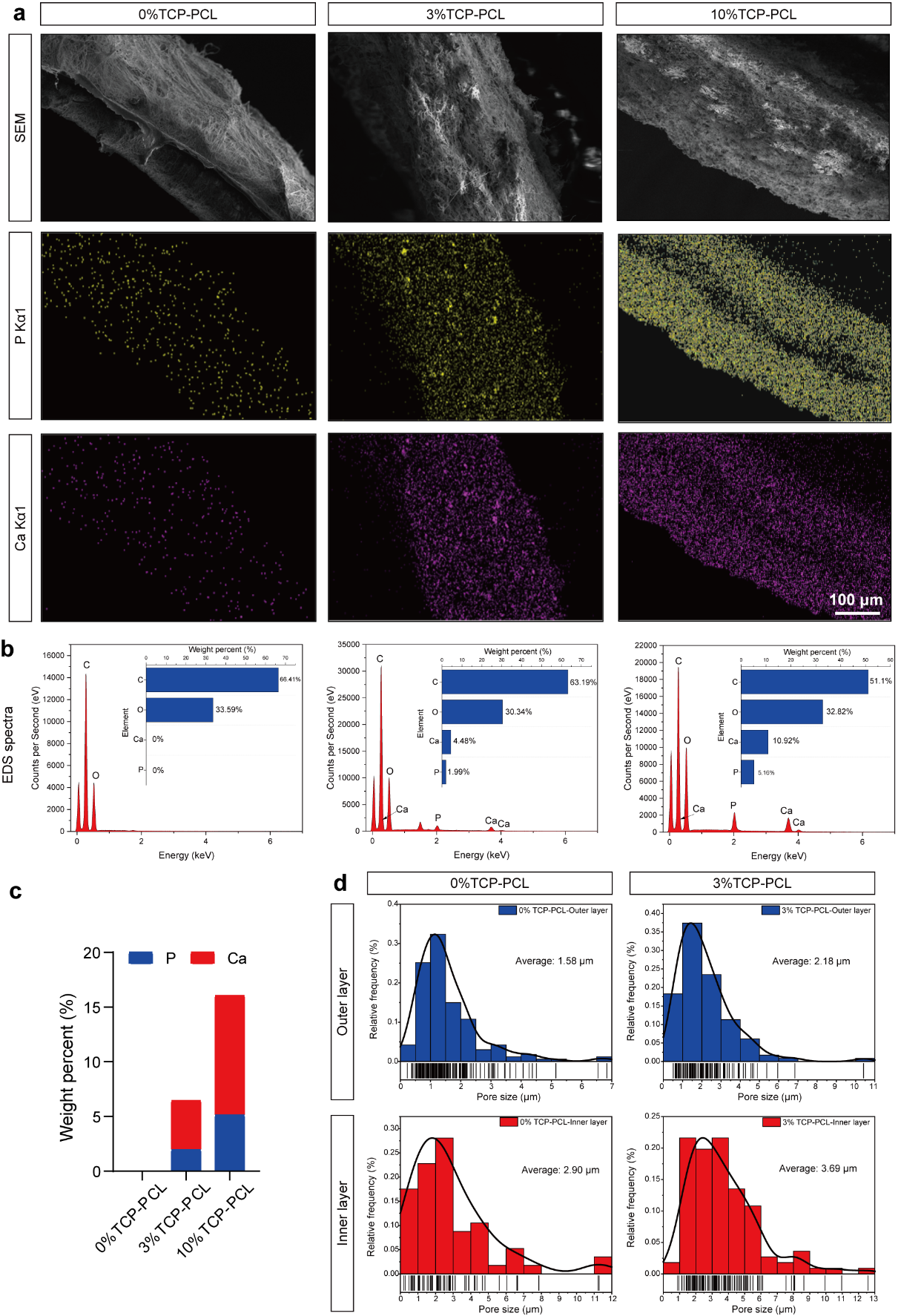


**Figure S3. Elemental composition analysis and pore size frequency distribution of biomimetic periosteum.** **a**-**c**, EDS spectra and quantitative analysis of biomimetic periosteum with different TCP concentrations. **d**, Pore size frequency distribution of inner and outer layers of 0% TCP-PCL and 3% TCP-PCL biomimetic periosteum. Scale bar: 100 μm.


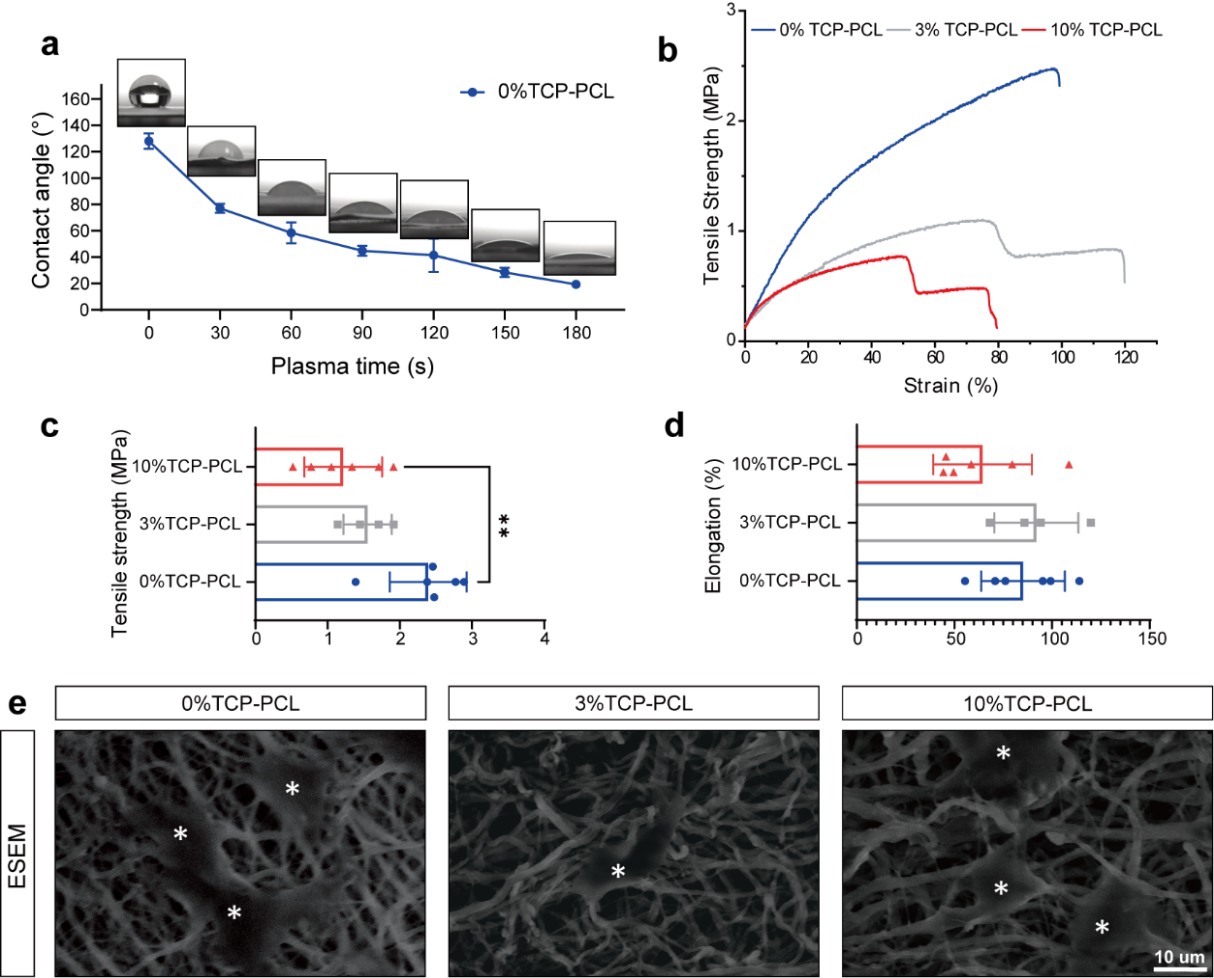


**Figure S4. Hydrophilicity assessment and biomechanical characterization of biomimetic periosteum.** **a**-**c**, Contact angle curves of biomimetic periosteum as a function of plasma treatment time. **b**, Stress-strain curves of biomimetic periosteum with varying TCP concentrations. **c**,**d**, Tensile strength and elongation at break of biomimetic periosteum across TCP concentrations. **e**, Environmental scanning electron microscopy (ESEM) images of vBR-MSCs (*) cultured on biomimetic periosteum with different TCP concentrations. Scale bar: 10 μm. Data are presented as the mean ± SD. *P < 0.05, **P < 0.01, ***P < 0.001, ****P < 0.0001, ordinary one-way ANOVA followed by Tukey’s multiple comparisons test.


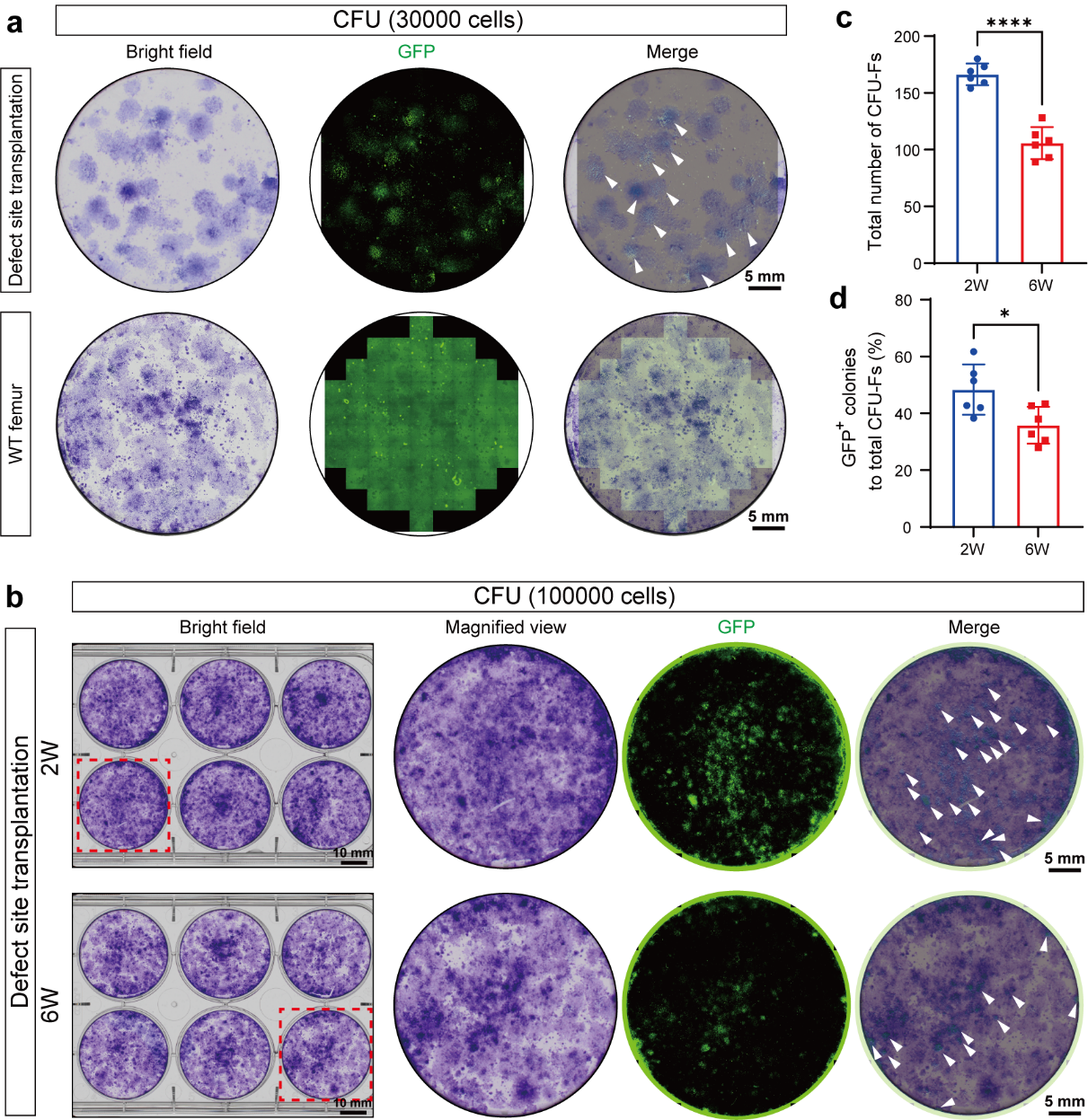


**Figure S5. Validation of strategy feasibility through allogeneic transplantation. a**, Representative images of CFU-F assays. Colonies were derived from cells isolated from femoral defects in wild-type (WT) mice 6 weeks after receiving GFP-labeled vBR-Bone transplants, compared with BMSCs isolated from native femurs of WT mice. Colonies formed by WT femur-derived BMSCs served as a negative control and showed no GFP signal, confirming the specificity of GFP^+^ colonies. **b**, CFU-F assays of GFP^+^ MSCs at 2 and 6 weeks after allogeneic transplantation. The magnified views on the right correspond to the areas indicated by red dashed boxes in the bright-field images on the left. White arrows: GFP^+^ colonies. **c**, Total number of total CFU-Fs. **d**, Percentage of GFP^+^ colonies among total CFU-Fs. Scale bar: 5 mm (**a**, right of **b**), and 10 mm (left of **b**). Data are presented as the mean ± SD. *P < 0.05, ****P < 0.0001, Unpaired, two-tailed Student’s t test.


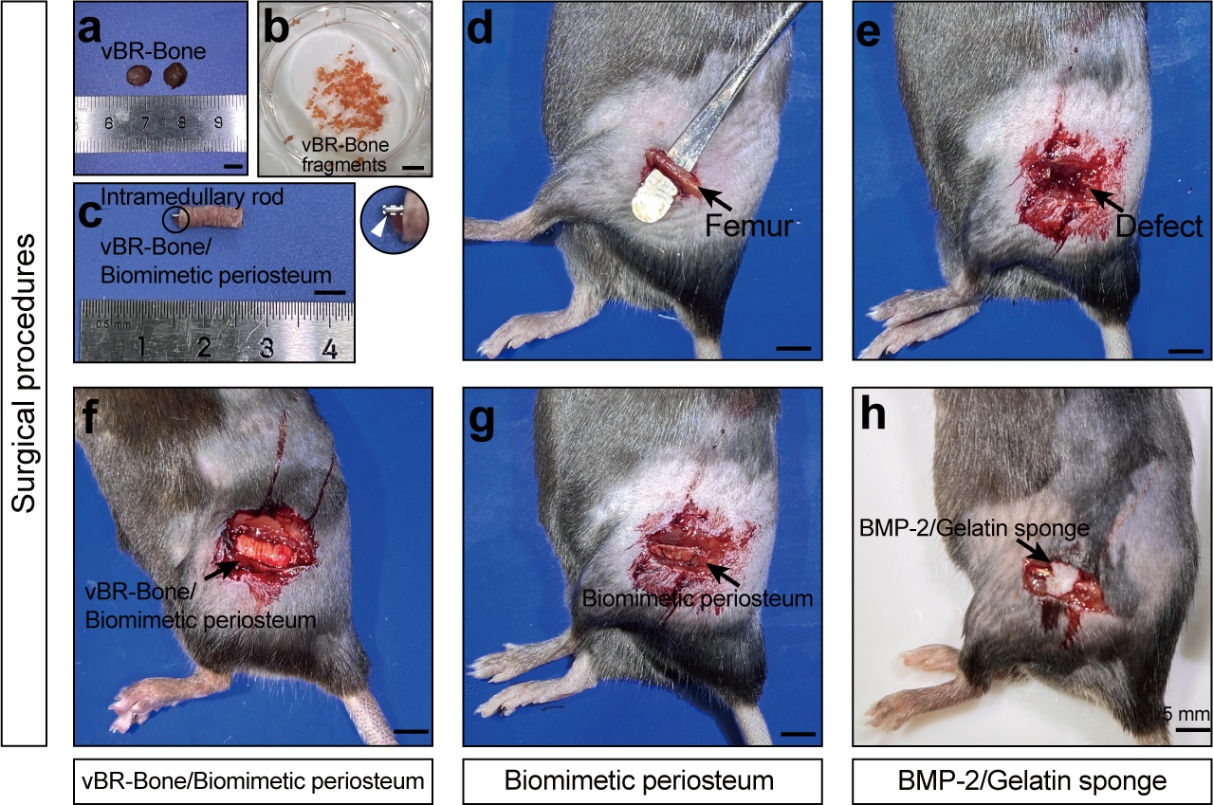


**Figure S6. Surgical procedures for segmental defect repair in experimental groups. a**,**b**, Harvested vBR-Bone was mechanically fragmented. **c**, vBR-Bone fragments combined with an intramedullary rod were wrapped in biomimetic periosteum to form the vBR-Bone/biomimetic periosteum composite, prepared for transplantation. White arrows: intramedullary rod. **d**, Femur exposure via skin/muscle dissection. **e**, Creation of 4-mm segmental defect by osteotomy. **f**, Implantation of vBR-Bone/biomimetic periosteum composite into the defect, followed by muscle/skin closure and povidone-iodine sterilization. **g**,**h**, Defect repair procedures using biomimetic periosteum alone or BMP-2/gelatin sponge in reference groups. Scale bar: 5 mm.


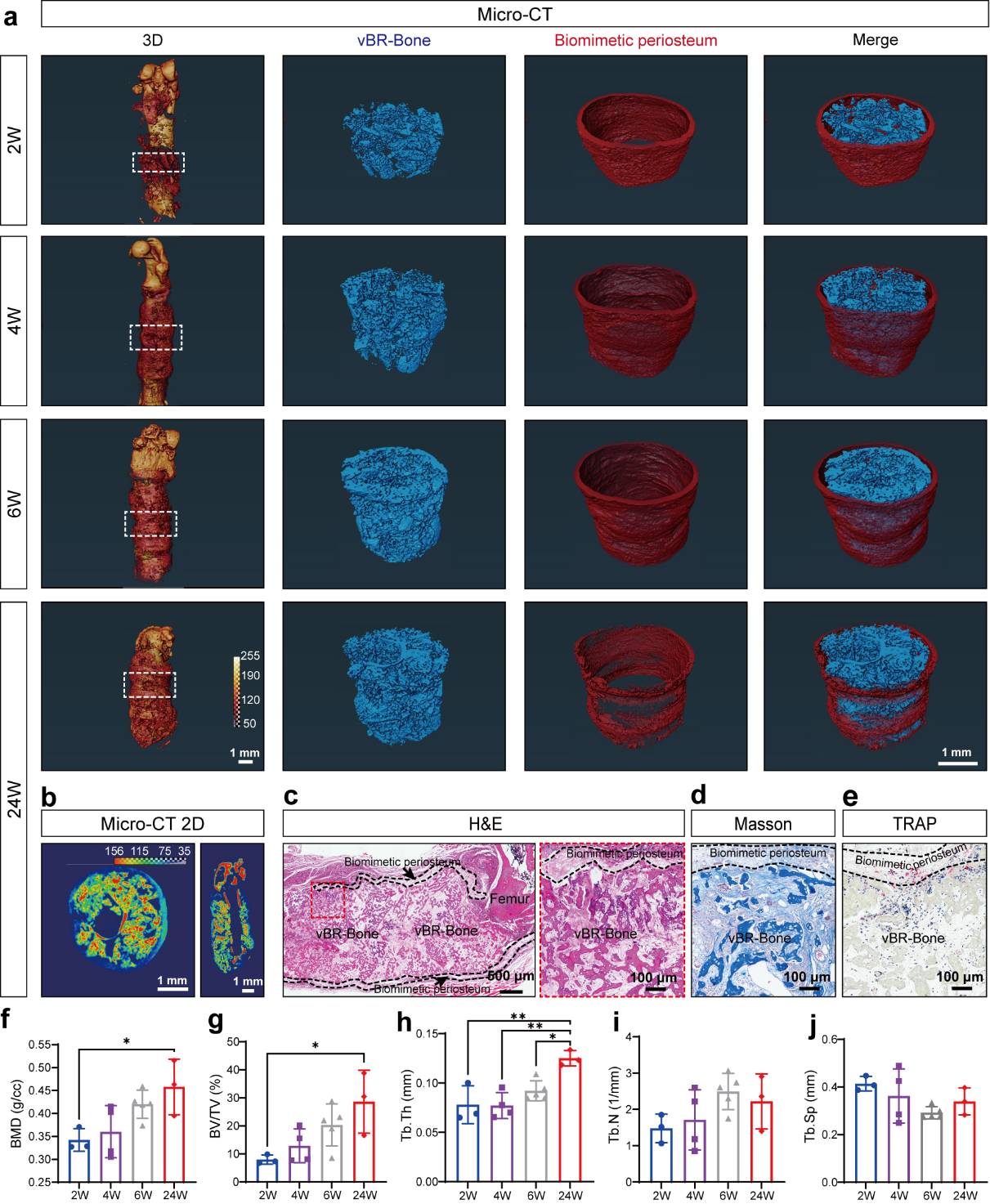


**Figure S7. Repair process of the reconstructed femur following vBR-Bone autologous transplantation.** **a**, Micro-CT 3D reconstruction images of the vBR-Bone/biomimetic periosteum treatment group at 2 ,4, 6, and 24 weeks after autologous transplantation into bone defects. **b**, 2D cross-sectional views of the vBR-Bone/biomimetic periosteum treatment group at 24 weeks after autologous transplantation. **c**-**e**, H&E, Masson, and TRAP staining of critical bone defect repair in the vBR-Bone/biomimetic periosteum treatment group. Scale bar: 1 mm (**a**,**b**), 500 μm (**c**) and 100 μm (magnified image of **c**,**d**,**e**). (**f**-**j**) Quantitative analysis of micro-CT (N = 3-5). Data are presented as the mean ± SD. *P < 0.05, **P < 0.01, ***P < 0.001, ****P < 0.0001, ordinary two-way ANOVA followed by Tukey’s multiple comparisons test.


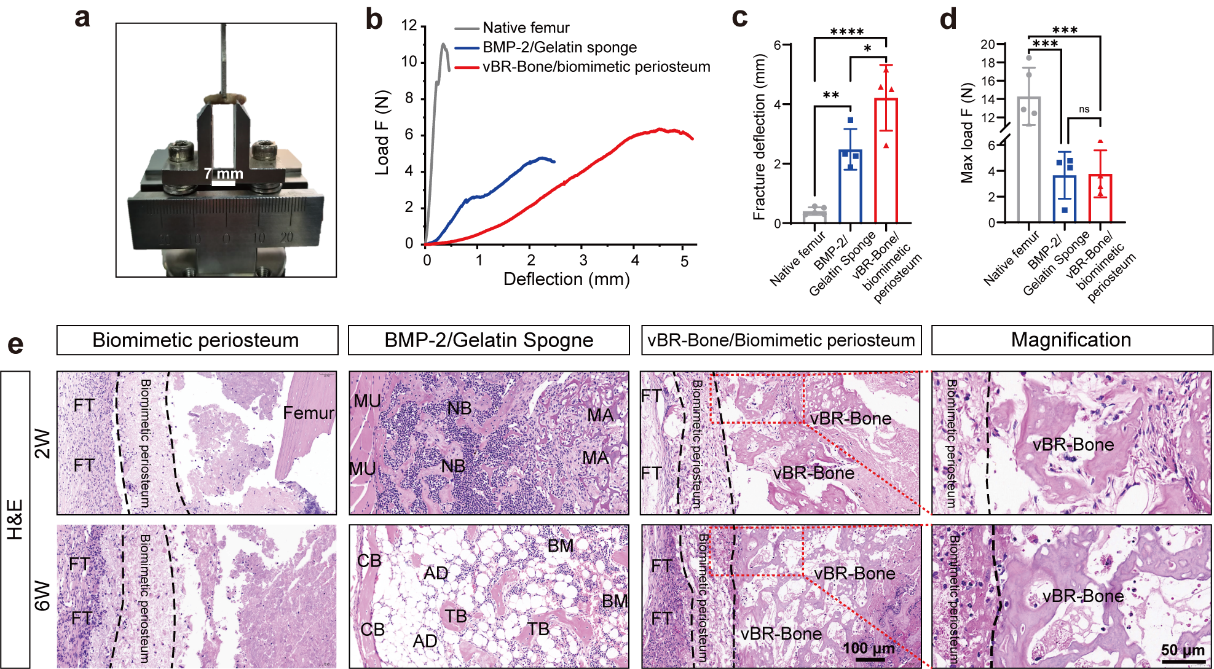


**Figure S8. Post-repair biomechanical tests and H&E staining of segmental defects.** **a**-**d**, Three-point bending tests and quantitative analyses of the BMP-2/gelatin sponge group and the vBR-Bone/biomimetic periosteum group at 2 and 6 weeks after transplantation into segmental bone defects, with the native femur as a reference (N=4). **e**, H&E staining of critical bone defect repair in the biomimetic periosteum treatment group, BMP-2/gelatin sponge treatment group, and vBR-Bone/biomimetic periosteum treatment group. Scale bar: 7 mm (**a**), 100 μm (**e**) and 50 μm (magnified images). Data are presented as the mean ± SD. *P < 0.05, Unpaired, two-tailed Student's t test.


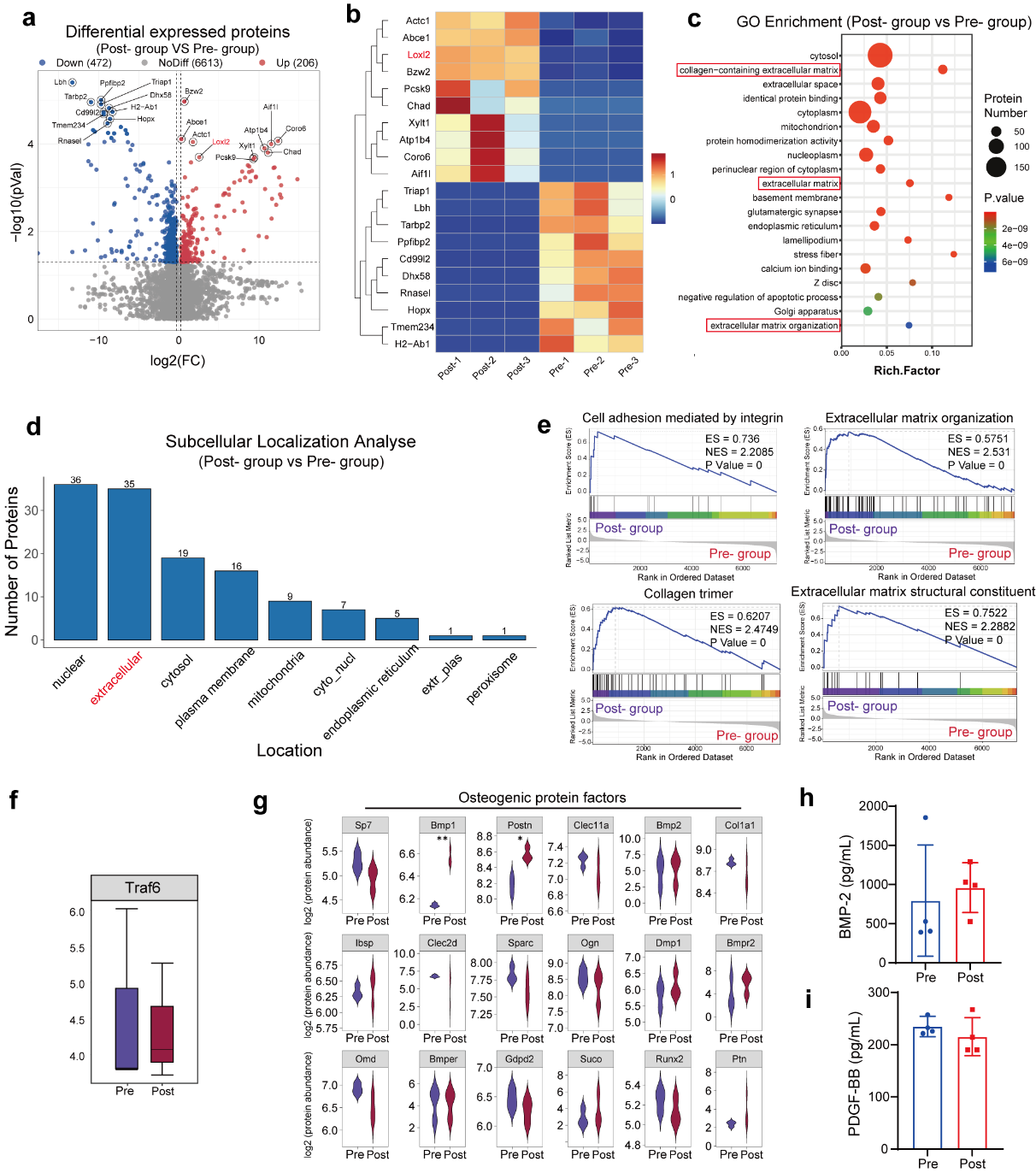


**Figure S9. Comparison of expressed proteins between the Post- group and Pre- group.** **a**, Volcano plot of differentially expressed protein profiles. **b**, Heatmap of differentially expressed proteins (top 20). **c**, GO enrichment analyses comparing the post- group vs. pre- group. **d**, Subcellular localization analyses comparing the post- group vs. pre- group. **e**, GSEA analysis of the post- group vs. pre- group. **f**, Violin plot depicting “Traf6” protein associated with the osteoclast differentiation pathway. **g**, Violin plots of osteogenic protein factors’ abundances. **h**,**i**, Elisa assays for BMP-2 and PDGF-BB (N = 4). Data are presented as the mean ± SD. *P < 0.05, **P < 0.01. Unpaired, two-tailed Student’s t test.


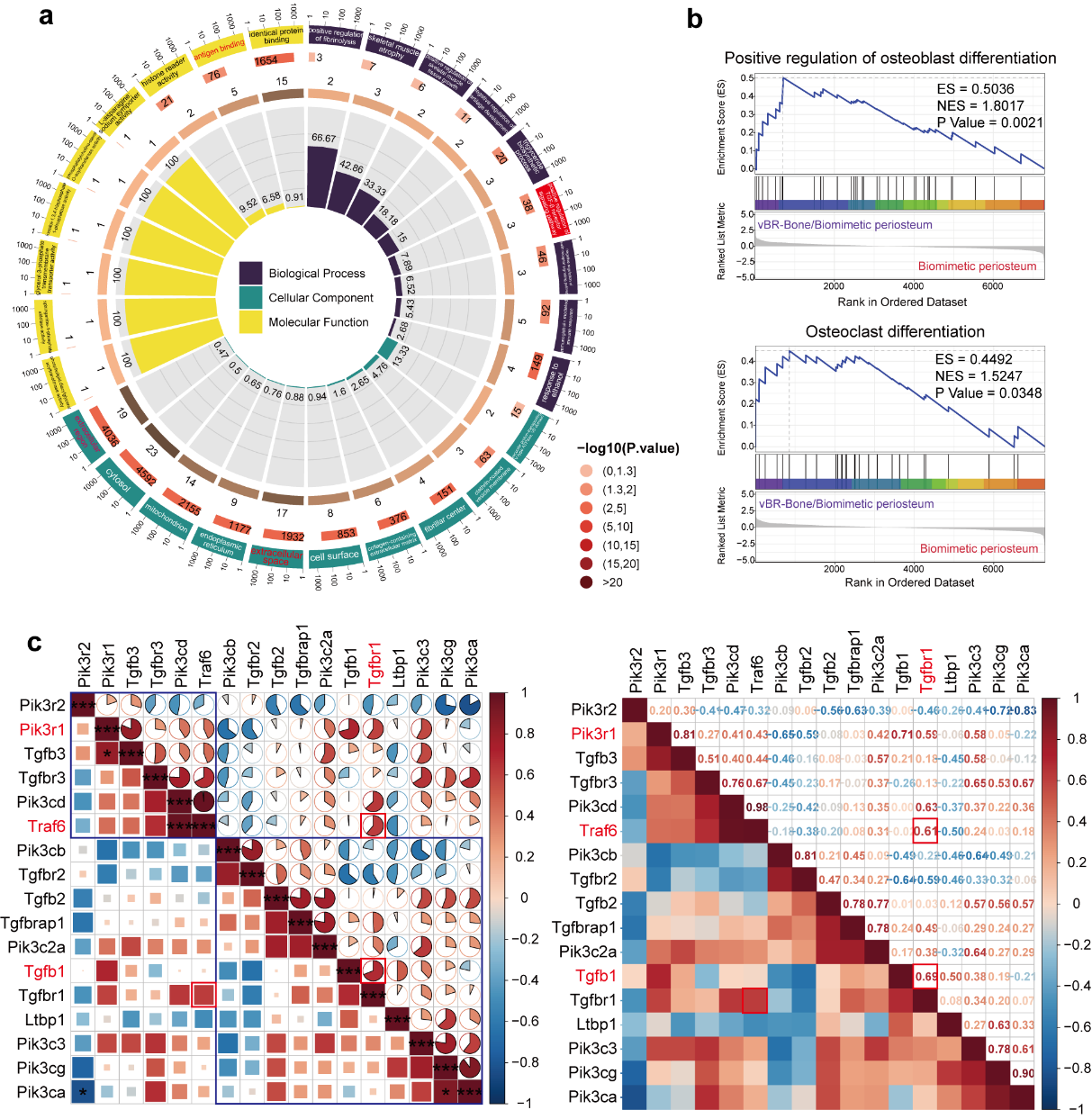


**Figure S10. Proteomic analysis between the vBR-Bone/biomimetic periosteum treatment group and the biomimetic periosteum treatment group.** **a**, GO enrichment circular plots across distinct biological categories. **b**, GSEA analysis of the vBR-Bone/biomimetic periosteum treatment group vs. the biomimetic periosteum treatment group. **c**, Correlation coefficient plot of TGF-β ligands, receptors, TRAF6, and PI3K subunits. Correlation method: pearson, *P < 0.05, ***P < 0.001. Unpaired, two-tailed Student’s t test.

**Table S1**

| GAPDH (Forward) | AAATGGTGAAGGTCGGTGTGAAC |
| --- | --- |
| GAPDH (Reverse) | CAACAATCTCCACTTTGCCACTG |
| OPN (Forward) | AGCAAGAAACTCTTCCAAGCAA |
| OPN (Reverse) | GTGAGATTCGTCAGATTCATCCG |
| COL1A1 (Forward) | TAAGGGTCCCCAATGGTGAGA |
| COL1A1 (Reverse) | GGGTCCCTCGACTCCTACAT |
| SOX9 (Forward) | GAGCCGGATCTGAAGAGGGA |
| SOX9 (Reverse) | GCTTGACGTGT GGCTTGTTC |
| Trp53 (Forward) | CAGTCTACTTCCCGCCATAA |
| Trp53 (Reverse) | GTCTCAGCCCTGAAGTCATAAG |
| BSP (Forward) | CCAAGAAGGCTGGAGATGCA |
| BSP (Reverse) | TCTGACCCTCGTAGCCTTCA |

**Table S1. PCR primers used in this study.**
